# Quarterly Periscope of Practical Medicine: Part I

**Published:** 1827-07

**Authors:** 


### Quarterly Periscope

OF

### PRACTICAL MEDICINE;

BEING

### The Spirit of the Medical Journals,

*Foreign and Domestic;*

WITH COMMENTARIES.

### PART I.

"Ore trahit quodcunque potest, atque addit acervo."

### 1. MM. BOUILLAUD AND CRUVELHEIR ON THE ANTERIOR LOBES OF THE BRAIN.*

We think it much more likely that the material organs of certain faculties will be ascertained by pathology than by experiments on living animals. And as this is the course which Professor Cruvelheir and Dr. Bland have pursued, with the view of solving the above problem in physiology, we shall dedicate a few pages to the subject in this place.

The loss of speech, observes M. Cruvelheir, may be caused in three ways—1mo. By loss of memory of *things*—2ndo. Loss of memory of *words*—3tio. The inability to *articulate sounds*. The first cause, he thinks, has been greatly over-rated, by confounding it with the two others:—though it is easily discriminated from them. Falls on the head, attacks of apoplexy, some bad fevers, &c. may induce the first condition, which is sometimes partial, sometimes temporary, sometimes permanent.

The obliviscence of *words* is a much rarer case, and may exist with perfect integrity of the intellectual functions. The absence of words by no means implies the absence of ideas. The examples on record are numerous, where men have forgotten the words of a language—substances, proper names—nay almost the whole of their words, whilst their intellectual faculties were sound. Finally, the loss of speech may exist without loss of memory of things. Men have been able to write with fluency and correctness, though they could not speak—and, strange to say, they have been able to execute all the other movements and

* "Has the faculty of articulating sounds its seat in the anterior lobes of the brain, as maintained by Dr. Gall?"

functions of the tongue, without the power of uttering an articulate sound. The following is a striking example.

*Case* 1. A man aged 65 years, of sanguine temperament, but lame, from repeated attacks of rheumatic gout, was suddenly seized with hemiplegia of the right side, and inability to pronounce words. Bleeding and various revulsives were used, and some power returned in the lower extremity. The mouth, which was much drawn to one side, recovered its proper position, in a degree, and the tongue could be pushed straight out. But he could pronounce no other words than " oui, non, mon Dieu !" and a few oaths which he had been accustomed to deal out very freely previously. His intellects were perfectly sound—he had lost no part of his memory—and was very greedy of the news of the day. He read books (without pronouncing the words) —played at cards with perfect correctness—laughed, wept, &c. but never without cause. Sometimes, forgetting his infirmity, he made violent attempts to speak, and at these periods could move the tongue in every possible direction ; but still without any success. Our author endeavoured to teach him to speak by monosyllables, but he was of too impatient a disposition to make any progress. He continued in this state four years, during which he enjoyed good health, and then died suddenly, when M. Cruvelheir was from home, and no dissection was made.

*Case* 2. A patient was received into the Hotel Dieu, who had been hemiplegiac of the *right* side for eleven months. He could only pronounce the monosyllables *oui* and *non*, although he appeared to be in full possession of his intellectual faculties. He died of acute pneumonia. On dissection, a considerable depression was observed in the middle part of the convexity of the *left* hemisphere. On cutting into this part, there was much resistance offered to the scalpel. They then came to a lamellated tissue, of yellow colour, infiltrated with serum, and forming a kind of cyst, with dense parietes, lined by a fibro-cellular membrane. This cyst or cavity occupied the centre of the *left* hemisphere. There was no disease in the anterior lobes of the brain.

The following case is curious and interesting in many points of view.

*Case* 3. A man was brought into the Hotel Dieu in 1820, offering the following phenomena:—Hemiplegia of the *right* side—loss of speech—great difficulty of swallowing. His intellects unaffected. M. Tavernier, the physician, went to the patient's house to learn the history of the case, and was informed by his wife that, eight years previously, her husband was seized with hemiplegia of the *left* side and great embarrassment of speech, immediately after a violent paroxysm of anger. His intellects, however, had not suffered. In the course of some months he recovered from this paralysis, the power of speech still continuing somewhat embarrassed. The present hemiplegia had come on the day preceding his entry into hospital, without any ostensible cause. All the usual means were employed at the hospital, but without success; and he died on the tenth day of his illness.

*Dissection.* The arachnoid covering the convexity of the hemispheres was rather thickened and injected. That covering the cerebellum was of a milky whiteness—plexus choroides very much injected. In the middle lobe of the left hemisphere, near its union with the anterior lobe, and exterior to the thalamus nervi optici of that side, there was found a clot of blood, the size of a nut, encircled by a yellow-red stratum of cerebral substance. The neighbouring portion of brain was softened to a small distance from the clot. The rest of the cerebrum was of its natural consistence. In the *left* lobe of the cerebellum, a cyst, half an inch in diameter, with well organized parietes, was discovered, filled with a yellowish serum.

This case presents us with one of those curious exceptions to a general rule, which we sometimes observe in apoplexy. The extravasation, in both attacks, was on the *left* side, though the paralysis was, in the first attack, on the left, in the second, on the right side of the body. There are, it is true, but few well-authenticated instances of the hemiplegia being on the same side as the sanguineous extravasation; but still there are some examples which cannot be questioned. We have, ourselves, seen two or three instances. They are very inexplicable.

*Case* 4. A merchant, aged 48 years, of bilious temperament, and of very active habits, experienced, without any visible cause, a great loss of power in the tongue, and distortion of the mouth. The physician who was called in, contented himself with prescribing pediluvia and frictions. Little amendment took place; and at the end of six months he experienced a second attack, accompanied by hemiplegia of the *right* side. Drastic purgatives, blisters, and frictions were the only means employed this time. The hemiplegia nearly disappeared, but the embarrassment of the tongue continued. M. Cruvelheir was now consulted:—there was no cephalalgia—no affection of the intellectual functions—moral sensibility greatly increased—high degree of irascibility—frequent fits, alternately, of laughing and crying—embarrassment in the movements of the tongue—mastication difficult, as also deglutition—articulation of sounds slow and difficult. In other respects, the digestion was good—the sleep tranquil—bowels constipated. This state continued unaltered for two years, at which period, he was occasionally affected with sudden diminution of power, sometimes in one side of the body, sometimes in the other. At these times the embarrassment of the tongue was augmented. Soon after this, he could not retain the saliva in the mouth—the mastication and deglutition became more and more difficult, and the articulation of words more embarrassed. In January, 1824, three years from the commencement of the illness, the appetite failed, the strength diminished, and swallowing became a matter of extreme difficulty. To these were added vomitings, at first at intervals, but afterwards constantly—fever. Yet the intellects were still free. He died suddenly in this condition. Before dissection, M. Cruvelheir prognosticated (we wish he had adduced more than his own assertion for this) that the seat of some of the cerebral lesions would be found in the tuber annulare, the peduncles of the cerebrum, or the upper portion of the medulla spinalis.

*Dissection.* The meninges were healthy, except that portion of the arachnoid covering the tuber annulare, the origin of the spinal marrow, and the optic nerves, where it was thickened, and of a dark colour. In the left hemisphere of the brain several minute morbid appearances were discovered, consisting of varicose dilatation of vessels, and small cysts, without contents. In one spot, however, on a level with the corpus callosum, and in the centre of the medullary matter of the left hemisphere, the scalpel met with a resisting body, which required as much force to cut through as the fibro-cartilaginous portion of the nose. It consisted of a hollow cyst, situated in the substance of the corpus striatum.

In the corpus striatum of the right hemisphere, there were two cysts filled with serum, and having yellow parietes. The tuber annulare was dense, and of a yellow colour throughout. In its centre was a depôt of blood not coagulated, the containing parts appearing as if torn. This depôt was the size of an almond. Near this was another depôt of ancient date, and with organized parietes. The lungs were sound—left side of the heart in a state of hypertrophy, the right side wasted and diminished. There were signs of chronic inflammation in the mucous membrane of the stomach, with abrasion of its villous structure.

In this examination our author found the vestiges of fifteen apoplectic attacks, the last of which took place in the tuber annulare, and occasioned death. The author anticipates a question that may be asked here—why did he not recognize the disease of the heart, during the patient's life? He excuses himself, by saying that the patient did not exhibit any symptoms of this disease, the pulse being regular, and no pain complained of in the region of the heart. This is rather a lame excuse; because, at that rate, no value is to be set on auscultation. If affections of the heart are expected to be discovered by the pulse and by the symptoms which the patient describes, the practitioner will be often deceived. Our author observes, that where the chambers of the heart are proportionally enlarged or dilated, little inconvenience will be felt in the functions of respiration and circulation. It is only where there is a disproportion in the morbid dilatation that the functions are disturbed. This is true only to a certain extent. An enlargement of the heart, however equally increased in all its chambers and parietes, is a serious evil, though not so incompatible with life as where one chamber is out of proportion to the other.

*Case 5.* An infant, four years of age, was presented to our author in the following condition:—General debility, so as to be unable to stand, though without any distinct paralysis—deglutition very difficult, particularly of liquids—articulation of sounds very slow—breathing slow and laborious, and it could only lie in a particular posture without danger of suffocation. In other respects, the intellectual powers were above par, and its embonpoint considerable. This state had continued three years, at which epoch it had a convulsion fit, occasioned, it was supposed, by a fall on the head. These convulsions had returned from time to time, and the child was thought to be epileptic. Professor C. looked upon the case as beyond the resources of art, and judged the tuber annulare to be the seat of the disease. Five or six months afterwards, this child died in a state of asphyxia, but without shewing any diminution of intellect till the last. On dissection, he found the corpora olivaria indurated to a cartilaginous consistence, but offering no other change. One of the cerebellic peduncles (he forgot to note which) participated in this induration. Every other part of the brain and cerebellum was perfectly sound. The spinal marrow was also sound.

*Remarks.* When it is recollected that the hypoglossi, glosso-pharyngei, and pneumo-gastric nerves arise about the corpora olivaria, we need not be astonished at the disturbed functions of those organs to which these nerves are distributed, viz. the lungs, the pharynx, tongue, &c.

The foregoing cases have proved that the loss of articulating power was the result of organic lesion of the *middle* lobes of the brain; the following cases are introduced to shew that the *anterior* lobes may be disorganized, without the loss of the said power.

*Case* 6. M. R. aged 42 years, of strong constitution, who had never complained of headache, or evinced the least disorder of intellect, became melancholy and peevish, without any apparent change of health. About a year after this, he had assisted at a public procession, with his bare head exposed to the sun, and became affected with violent cephalalgia, which continued several days, taking a periodical form. The cinchona was administered, and in six weeks the head-aches ceased. Complete amaurosis then took place, for which galvanism was ineffectually employed. Fever supervened, and death closed the scene, but still without any disorder of intellect till the last, or any difficulty of speech.

On dissection, the anterior lobe of the right hemisphere was found completely disorganized, being one vast pouch or cyst, containing a pulpy homogeneous matter. The thalami nervorum and other portions of brain were sound; but the optic nerves themselves were shrunk.

*Case* 7. N. aged 24 years, of robust constitution, and florid complexion, consulted our author on the 12th Dec. 1821, on account of an œdema of the left cheek and eyelids. There was a kind of chemosis of that eye, and severe supra-orbital pain, and some fever. These symptoms had continued for several days, and had been preceded by some smart febrile paroxysms. He had received no fall or other injury. Leeches and other antiphlogistic means were employed. By these the symptoms were relieved, and our author lost sight of the patient for some days. On the 30th Dec. M. C. was again sent for, and found his patient remarkably emaciated for so short a time. He still had pain in the left eye, which was very sensible to light. The pulse was natural, but he had vomiting after the ingestion of food or drink. On the 1st January, 1824, M. Cruvelheir was astonished at the slowness, inequality, and smallness of the patient's pulse, and immediately suspected some organic disease of the brain. The patient could not now use any mental exertion, and desired to be left alone and in the dark. He was bled from the feet, and some other means were used, but the malady increased. He saw double with the left eye; had strange noises in his ears—slow breathing—sighing—excessive susceptibility to the slightest moral or physical impression. He died on the 14th of January.

*Dissection.* The anterior lobe of the left hemisphere was one vast abscess, filled with thick, greenish pus. There was matter of the same kind under the arachnoid, covering the pons varolii and origin of the spinal marrow. The parietes of the abscess were formed of a membrane a quarter of a line in thickness, whose internal surface was red, rugous, and granulated. There was softening of the cerebral substance around the parietes of the purulent depôt. This man could pronounce his words distinctly to the last, and yet one of the anterior lobes was disorganized.

Our author concludes, from these facts, that it is not proved that the faculty of articulating sounds resides in the anterior lobes of the brain. However this may be, we consider the foregoing cases as interesting in a pathological point of view, as well as in a physiological.—*Nouv. Bibliot.*

---

### 2. KINO IN DROPSY.

There is a curious case recorded in the Feb. No. of the Medical and Physical Journal, by Dr. Paul, of Elgin, which is worthy of notice. The patient was a colonel in the Royal African corps, who had returned from the coast of Guinea, in October, 1825, in a desperate state of health. He had irritative fever, great prostration of strength, effusion in the abdomen, anasarca of the ankles and feet, tenderness in the region of the liver, great irregularity of bowels, emaciation. The abdominal effusion increased, in despite of every species of diuretic, including mercury, and, on the 26th December, it was necessary to draw off eighteen pints of fluid. Diuretics were again tried, but the abdomen filled, and, by the 24th Jan. the operation again became indispensible. He was now so weak, that they thought the Colonel would have died among their hands. Sixteen pints were drawn off. The fluid in the abdomen collected again as rapidly as ever. As diuretics had completely failed, and the stomach nauseated them, Dr. Paul determined on a novel, and, as it proved, a fortunate remedy. Dr. P. prescribed half an ounce of tincture of kino in port wine daily. It was commenced on the 4th Feb. and continued a few days, when the dose was increased till he took an ounce daily of the tincture, with full a pint of port. This plan was continued till the first of April, after which an ounce served for three days. When he began this plan, the abdomen was nearly as full as before tapping. In eight days his appetite began to improve, the strength increased, the pulse fell in frequency. The abdominal swelling remained stationary for a time, and then decreased, till he was able to walk about, and even to undertake a journey to London. Since that period, he has improved wonderfully in health, and, although there is still some abdominal effusion, it is not unreasonable to expect that it will be altogether removed. Every eight or ten days, he takes three grains of calomel at night, and some tincture of rhubarb in the morning. The history is communicated in a letter to Sir James M'Grigor, (who saw the patient) and is, therefore, authentic.

This case proves that all dropsies are not dependent on inflammation, which is the ultra-doctrine of the day. There are dropsies of debility; else, why should we generally find œdema of the ankles so common in all weak states of the body. We think it is probable that any good tonic, as the quinine, with generous wine, would have produced the same beneficial change, in the above case, as the kino. The event is well worthy of record, as a check to the doctrine which almost exclusively attributes serous exhalation to inflammatory action of the vessels carrying red blood.

---

### 3. DOUBLE HEPATIC ABSCESS.

The liver is a viscus whose organic sensibility does not appear to be of the most acute kind, especially as compared with the stomach, duodenum, or small intestines. Its functions are intimately connected with health—but its derangements may amount to a great extent, functional or structural, before life is endangered. Its sympathies are numerous, but, like its sensibility, they are not of the most vivid character. Abscesses in this organ often break down and destroy a great portion of its structure, with comparatively little disturbance of the general system, especially if the suppurative process be slow. The prognosis, however, in all hepatic abscesses is grave—often too grave, as the following case, recorded by M. Cavalier, in the January number of the Revue Médicale, will shew.

*Case.* In August, 1825, Joseph Revel, a young peasant, was seized with acute hepatitis, of unequivocal character. The fever was intense, the pain of the right side, stretching to the shoulder, violent; but there was no vomiting—no jaundice. The treatment was rigidly antiphlogistic, and by the end of the month the patient was convalescent. He returned to his occupations, but still he felt a deep-seated and obscure pain in the right hypochondrium. Towards the end of September, after some strenuous corporeal exercise, this pain assumed a more acute character, and was accompanied by some febrile movement, which obliged him to give up work. In a few days afterwards, an indolent tumour was perceived in the right side of the back, between the 7th and 10th ribs, which, by the 20th October, had acquired the size of a child's head. For some reasons, not clearly stated, the caustic potash was employed instead of the knife, to open this vast abscess. The slough came away on the 24th October, and about a pint of matter, of a reddish colour, with flocculi partly dissolved, the whole having the appearance of lees of wine. The discharge was also pretty profuse on the dressings. The fever now took a hectic character, and emaciation rapidly advanced. Nourishing food and the bark were prescribed. He was going on from bad to worse, when, on the 16th November, M. Cavalier was called to the patient in great haste, as he was said to be dying by vomiting of blood. On arrival, it was clearly seen that the patient was not vomiting blood, but the same kind of material which had been discharged from the abscess in the back. He was nearly suffocated by this new discharge: but it gradually lessened, though it still continued more or less to be expectorated for three weeks afterwards. The original abscess continued to discharge all the time, apparently unaffected by the new formation. Injections thrown into the hollow of the dorsal abscess did not pass into the lungs, or occasion any taste in the mouth, when bitter infusions were thrown in. These circumstances led to the conclusion, that the abscesses were distinct in the liver. The patient continued in a deplorable condition for about eighteen days, when the expectoration greatly diminished—the cough subsided, and, to the great surprize of our author, they both finally ceased, while the hectic fever diminished, and the general health of the patient somewhat improved. Nevertheless the dorsal abscess became fistulous, and still furnished an abundant discharge. In this condition the patient was received into the hospital, (Draguignan) with little hope of recovery. A probe passed several inches into the fistula—the fever continued stationary—the digestion was impaired, and although there was not actual icterus, the skin had assumed a very unhealthy sallow tint. Astringent injections were used, and nourishing food, with tonics, were continued. In the course of a month, the probe passed less profoundly into the fistula. In another month a diarrhœa came on, and the fever diminished. By the month of June, the abscess had closed, and, after this, the convalescence went on to complete recovery.

We shall only remark, on this case, that, in acute hepatitis, the cure should not be trusted entirely to bleeding and purging, as it generally is in this country. The glandular structure requires to be emulged by mercurials, while inflammatory action is controlled by blood-letting and the usual antiphlogistics. It was probably owing to the want of this auxiliary treatment, in the above case, (for in France mercury is rarely thought of in acute inflammation) that the abscess formed in the liver in spite of sanguineous depletion.

---

### 4. CATARRHO-RHEUMATIC OPHTHALMIA.

[Glasgow Eye-Infirmary.]

We have followed Mr. Mackenzie through his divisions of ophthalmic inflammation, and we are not among those who have taunted him with *German* minuteness in his distinctions. We believe, on the contrary, that *minute attention* to the phenomena of diseases, as modified by the structures which they assail, and the causes which produce them, is the only sure means of arriving at accurate diagnosis and successful practice. In answer to some queries which had been addressed to our author from London, as to what he meant by *rheumatic* ophthalmia, Mr. M. replies, *first*, that he means simply inflammation of a fibrous tissue of the eye, (the sclerotica) and of surrounding parts of similar texture, excited by atmospheric changes. *Secondly*, that this inflammation does not differ from chronic inflammation *in kind*, on account of the supposed rheumatic diathesis, but merely on account of the structure and functions of the part attacked. *Thirdly*, he has never known a metastasis of rheumatism from another part of the body to the eye, as frequently takes place to the heart. "In all cases of rheumatic sclerotitis which I have witnessed, the disease was primary, whether in rheumatic or in non-rheumatic subjects—never metastatic." *Fourthly*, he has taken, on trust, from Beer and Wardrop, the term "rheumatic ophthalmia," lest he should shock the reader by a new name; yet "sclerotitis atmospherica" would, he thinks, be a truer appellation. The inflammation in question, however, resembles rheumatism in its exciting causes, its accompanying pain, its exacerbations, and its cure. He thinks it has probably been less generally recognized as rheumatism, because the common seat of the latter, in general, is hidden from our view; whereas the disease under consideration attacks a structure which is covered only by a thin semi-transparent membrane, and is, therefore, exposed to direct examination.

But to return. In two former communications, our ingenious author described the pure catarrhal, and then the pure rheumatic ophthalmia, illustrated by cases treated publickly in the Glasgow Eye-Infirmary. He now proceeds to the catarrho-rheumatic ophthalmia.

In this disease, the conjunctiva and sclerotica are attacked simultaneously—the former by catarrhal, the latter by rheumatic inflammation. The symptoms, then, like the pathological conditions of the two diseases, are conjoined. If these be *German* distinctions, observes Mr. M. they are natural distinctions—anatomical distinctions—physiological distinctions—practical distinctions, of the greatest importance to all who have eyes to be cured, or who wish to cure the eyes of others.

Thus, on examination, we find the external covering of the eye liable to profluvial or muco-purulent ophthalmia—the fibrous texture beneath subject to rheumatic inflammation. In some ophthalmies we find one membrane affected, in others, the other, and, in many cases, both. Hence, there are three sets of cases occasionally presented to our view, the symptoms of which are so strikingly marked, that they cannot be overlooked by any practitioner of the least observation and attention, to whom they are once pointed out. They require different methods of cure. If discriminated, they are easily subdued—if confounded, they are apt to leave the eye permanently injured, or even deprived of sight. Under the three heads of symptoms, causes, and treatment, Mr. Mackenzie has laid down his descriptive, etiological, and therapeutical observations, in language so precise, that we are unable (and this is not a very common case) to condense it without loss to our readers. We shall, therefore, offer an extract, rather than an abstract, on this occasion.

"1. As both conjunctiva and sclerotica are affected in this disease, the symptoms are both more complicated, and also more various, than those of the unmixed conjunctivitis and sclerotitis, formerly described.

"2. The sense of roughness, which is compared by the patient to the feeling of sand between the eyelids and eyeball, and the secretion of purulent mucus and purulent meibomian fluid, are sufficiently indicative of the part taken in this disease by the conjunctiva. The nocturnal accession of racking circumorbital pain marks the affection of the fibrous sclerotica, the surrounding periosteum, and the neighbouring temporal fascia.

"3. In some cases of catarrho-rheumatic ophthalmia, the conjunctivitis is severe, the sclerotitis slight; but more frequently the sclerotitis is severe, the conjunctivitis not so considerable.

"4. In this disease the conjunctiva and sclerotica are attacked simultaneously. Occasionally it happens, in the course of pure rheumatic ophthalmia, that the patient, from some new exposure, becomes affected also with catarrhal conjunctivitis, as in the case of Mary Scott, page 44. More rarely does an attack of rheumatic sclerotitis supervene on catarrhal ophthalmia. But in catarrho-rheumatic ophthalmia, both membranes appear to be attacked at once, in consequence of the influence of one and the same exciting cause.

"5. In this disease, the redness is evidently both conjunctival and sclerotic. Under the moveable network of the conjunctiva, we perceive the immoveable zonular inflammation of the sclerotica. In pure catarrhal ophthalmia, the sclerotica, no doubt, partakes in the inflammation of the contiguous tunic, but no paroxysms of rheumatic pain are present: the sclerotica, suffers sympathetically, not primarily. In pure rheumatic ophthalmia, also, the conjunctiva is reddened, from contiguous sympathy with the structure which it invests, just as the skin is reddened over a joint suffering from acute rheumatism; but neither the conjunctiva in the one instance, nor the skin in the other, is the seat of the primary disease. Besides, in pure rheumatic ophthalmia, the conjunctiva betrays no marks of profluvial disease.

"6. Chemosis, or inflammatory œdema of the sub-conjunctival cellular substance, is by no means an uncommon attendant on catarrho-rheumatic ophthalmia. When it does occur, it hides from our view the sclerotic redness.

"7. The discharge from the conjunctiva in this disease is never profuse, and seldom opaque. It amounts, in general, rather to a mere increase of mucus, than a flow of pus.

"8. The eyelids adhere together in the morning, from the inspissated meibomian secretion. Not unfrequently they are also externally red and swollen.

"9. Considerable intolerance of light and epiphora attends this ophthalmia, in all its stages; but especially in those cases where the structure of the cornea is affected.

"10. The conjunctival pain, which is compared to the feeling produced by sand between the eyelids and eyeball, is felt most in the morning, or when the eyelids are moved. The sclerotic pain is nocturnal, and observes the same periods of renewal, violence, and abatement, which I have noticed in my paper on Rheumatic Ophthalmia. The conjunctival pain is referred to the surface of the eye, and sometimes to the forehead. The sclerotic pain is circumorbital.

"11. In this disease, the cornea is extremely apt to suffer from ulceration, and from effusion of pus between its lamellæ. Indeed, there is no ophthalmia to which adults are exposed, in which ulcer of the cornea and onyx are so frequent, as in the catarrho-rheumatic. If this disease is neglected for eight or ten days, and especially if the patient be far advanced in life, we almost uniformly meet with one or other, and not unfrequently with both of these symptoms.

" 12. The ulcer is peculiar. It spreads over the surface, rarely penetrating deeply into the substance, of the cornea. It generally cicatrises without leaving any opaque speck, the cornea remaining merely irregular, as if part of it had been hacked off with the lancet; and of course vision, from imperfect refraction, is confused. Professor Beer and Mr. Wardrop have described this kind of ulcer as attendant on pure rheumatic ophthalmia, but I have never seen it except in catarrho-rheumatic cases. Professor Beer mentions that it originates in a phlyctenula, but I have never had an opportunity of seeing any appearance of this kind. If the case continues to be neglected, or if it be mistreated, this ulcer ceases to be superficial; the substance of the cornea is more deeply attacked, and opaque leucoma will be the result.

" 13. Onyx, or effusion of pus between the lamellæ of the cornea, is the most alarming of all the symptoms of this ophthalmia. It generally commences at the lower edge of the cornea, in shape like the white spot at the root of the nails, convex on its upper edge, gradually increasing, mounting upwards, separating the lamellæ more and more between which it is effused, and greatly adding to the sufferings of the patient. It reaches not unfrequently to such a height as to implicate more than half of the cornea. The pus of an onyx in catarrho-rheumatic ophthalmia is very rarely absorbed. The cornea becomes ulcerated over the centre of the onyx; the pus is evacuated; the ulcer penetrates through the posterior lamellæ of the cornea; the aqueous humour escapes; the iris falls forward into contact with the ulcerated cornea; in nine cases out of ten, these parts adhere together, and the result is partial or total staphyloma.

" 14. As the onyx goes on advancing, there is commonly also an effusion of lymph going on in the pupil: the pupil becomes, first of all, less vivid in its motions; the colour of the iris changes; the pupil becomes hazy, contracts as the onyx increases, and may at last be obliterated.

" 15. In some cases, the onyx is accompanied by hypopium, or effusion of pus into the anterior chamber. In other cases, the onyx bursts first into the anterior chamber; false hypopium is thus produced, and ultimately the cornea gives way.

" 16. If luckily the matter of an onyx be absorbed, albugo remains for a considerable time, but gradually diminishes, and may ultimately almost entirely disappear. If onyx is dispersed by the cornea giving way, leucoma is the result, and never entirely disappears. Staphyloma cannot result, unless the iris and cornea have become partially or totally adherent. Mr. Wardrop remarks, that partial staphyloma generally affects the inferior half of the cornea. The reason is, that partial staphyloma is commonly the consequence of onyx, which in nine cases out of ten takes place at the lower edge of the cornea.

" 17. In catarrho-rheumatic ophthalmia, the pulse is generally quick and sharp; the tongue white, and mouth ill-tasted. The nocturnal pain completely prevents sleep, till about sun-rise. Catarrh sometimes attends, and adds to the febrile symptoms.

" 18. We generally find that the rheumatic symptoms yield first to treatment; the catarrhal continuing for some days longer. But in some cases I have observed the reverse: the circumorbital pain continuing in a slight degree after all the catarrhal symptoms were gone.

" CAUSES.

" The causes of catarrho-rheumatic ophthalmia appear to be similar at-

" * Morbid Anatomy of the Eye, vol. i. p. 106.

mospheric influences to those formerly enumerated as giving rise to catarrhal, and rheumatic ophthalmiæ. Amongst the poor, the disease may in general be traced to cold, to which the patients have been exposed, particularly during the night, from deficient clothing and want of proper shelter. Like other inflammatory and rheumatic affections, it is more prevalent during north-easterly winds.

Professor Beer thought that cold draughts of air,* playing upon the eye, excited rheumatic ophthalmia; and that foul air† caused catarrhal ophthalmia. According to this view, air at once corrupted and impelled with force against the eye, especially when the head is covered with perspiration, will be the most likely cause of catarrho-rheumatic ophthalmia.

"In 1805, at Riding-street Barracks, nearly twenty miles to the interior of Romney Marsh, the second battalion of the 52d Regiment appears to have suffered severely from catarrho-rheumatic ophthalmia. Dr. Vetch attributes the severity of the disease in that situation, and the intermittent form of some of the symptoms, to the influence of the marsh.‡

"That the discharge from the conjunctiva in catarrho-rheumatic ophthalmia, if applied to the conjunctiva of a healthy eye, will excite a puro-mucous conjunctivitis, is extremely probable, and is supported by such facts as I have recorded in Cases XII. and XIII. of a former communication.§ That catarrho-rheumatic ophthalmia can arise from contagion, is extremely improbable. In such cases as that of Coleman and her child, (Case VII. of the present paper,) both patients had been exposed, we may conclude, to the same exciting cause; and, while the one caught catarrhal ophthalmia, the other was seized with the catarrho-rheumatic form of this disease.

"Professor Beer mentions that catarrho-rheumatic ophthalmia sometimes occurs in children, and still more frequently in old persons, along with suppression of urine. But he seems to reject the conclusion of some, that this was any thing more than a mere coincidence; and he gives us no hope that diuretics would be peculiarly serviceable, even though they restored the secretion of urine.¶

"We meet with catarrho-rheumatic ophthalmia much more frequently in old persons than in the young or middle-aged.

"TREATMENT.

"The successful treatment of this disease does not depend so much on any new remedies, as on a proper selection of some of the means formerly recommended, either for the catarrhal or for the rheumatic ophthalmia.

"1. Venesection. This appears to be as necessary in the catarrho-rheumatic as in the pure rheumatic cases; and is attended by as remarkable relief to all the symptoms, especially to the circumorbital pain. According to the severity of the case, and the age and constitution of the patient, from ten to thirty ounces of blood may be taken from the arm; and the same quantity on the day following, if the symptoms are not greatly relieved.

"2. Leeches to the temple are also highly useful, particularly when applied soon after venesection.

---

"* Eine kalte Zugluft."

"† Ein zersetzer verdorbener Luftkreis."

"‡ See Vetch's Account of the Ophthalmia which has appeared in England since the Return of the British Army from Egypt, Lond. 1807, p. 20. Also Dr. A. T. Thomson's Remarks on Acute Rheumatism, in this Journal for February, p. 123."

"§ Vol. lvi. p. 329."

"¶ Beer's Leitfaden, vol. i. p. 310."

"3. Scarification of the conjunctiva of the eyelids proves useful in cases of chemosis; but produces comparatively little effect, unless practised in the manner described at page 324, vol. lvi.

"4. Calomel and Opium. The same good effects are derived from this combination in this ophthalmia, as in the pure rheumatic. The dose, and the length to which the calomel should be pushed, are the same. See p. 41.

"5. Opiate Frictions on the forehead and temple, about an hour before the expected attack of circumorbital pain.

"6. Belladonna, so as to keep the pupil dilated.

"7. Blisters behind the ear, or to the nape of the neck.

"8. Purgatives; such as a brisk dose of calomel and jalap at the beginning, and a gentle laxative every morning during the course of the disease.

"9. Sudorifics; such as Spiritus Mindereri, diluent drinks, the warm pediluvium, and a flannel under-dress.

"10. Tonics; such as Cinchona and the Mineral Acids, in the chronic stage of the disease. Under these heads, I have nothing to add to what is stated at pages 41 and 42 of this volume, and at page 325 of vol. lvi.

"11. Solution of Nitrate of Silver. As in the catarrhal, so in the catarrho-rheumatic ophthalmia, the solution of from two to four grains of nitrate of silver in one ounce of distilled water, dropped upon the conjunctiva once a-day, relieves the feeling of sand, and speedily removes the other symptoms of conjunctivitis. This application, however, has no effect on the sclerotic part of the disease; and I should conceive it a very dangerous mistake to trust to this remedy almost alone, as we may safely do in pure catarrhal ophthalmia, and to neglect the appropriate means for reducing the attendant inflammation of the sclerotica. See Mr. Melin's Report, in this Journal for September, 1824.

"12. Vinum Opii. Before the catarrhal part of this disease is subdued by the solution of nitrate of silver, this remedy rather aggravates the symptoms. After the conjunctivitis and the acute sclerotitis have yielded, it operates favourably, as in the chronic stage of the pure rheumatic ophthalmia; affording thus a good illustration of the remark of Boerhaave—'Nullum ego cognosco remedium nisi quod tempestivo usu fiat tale.'

"13. Collyrium Muriatis Hydrargyri, one grain to eight ounces, to be used milk-warm three or four times a-day.

"14. Unguentum Præcipitati Rubri, smeared along the edges of the eyelids at bedtime. These I employ as part of the treatment suitable for the conjunctival part of the disease, according to the directions given at pages 325 and 336 of vol. lvi.

"15. With respect to the treatment of onyx, I would recommend the lancet not to be used for evacuating the purulent fluid effused between the lamellæ of the cornea. In every case in which I have evacuated the matter with the lancet, partial or total staphyloma has been the result. In Ferrie's case, (Case VIII.) I left the matter to itself, and certainly no case could be more alarming in its progress, nor more unexpectedly happy in its results. I attributed the success which attended this case in a great measure to the sorbefacient influence of the calomel over the effusion into the pupil,—to the continued use of belladonna,—and to the gradual preparation of the cornea by nature for its giving way, and for its healing up; a preparation which would probably have been entirely defeated, had I ventured, as I had done in a number of previous cases, to open the onyx with the lancet." 300.

For a selection of cases illustrative of the foregoing descriptive and didactic matters, we refer to the April Number of the Medical and Physical Journal.

### 5. ON ALUM IN DIPHTHERITIS, OR MALIGNANT CROUP. BY M. BRETONNEAU.

Our readers will remember that, in our Number for October, 1826, page 419, *et seq.* we gave an extended analysis of Dr. Bretonneau's work on Croup, Angina Maligna, Angina Gangrenosa, &c. in which were shewn the inefficacy of depletion, and the good effects of certain local applications to the tonsils and fauces, before the peculiar or croupal exudation had spread down the larynx.

The same author has published a paper in the January Number of the Archives Generales, containing some further notices of the disease, and of the use of alum as a remedy. This substance has been recommended since the time of Areteus, in the various forms of inflammation and ulceration of the throat. At the close of M. Bretonneau's large work, he adverted to two cases of angina maligna, where alum was used, and apparently with good effects. Since that period he has had more frequent recourse to the remedy, and with success. We shall glance at some of the facts brought forward in support of the topical application of alum in this disease.

In the beginning of July 1826, a malignant angina broke out at Villandry, four leagues from Tours. A man had already fallen a victim to the disease, on the fourth day; and his wife becoming affected, M. Bretonneau was summoned to her assistance. This female was 21 years of age, of florid complexion, and had just weaned her child. When she first complained of her throat, an emetic had been administered. In the night, deglutition became painful, and next morning, one of the tonsils was covered with lichenoid concretions, and was much tumefied, as were the lymphatic glands on that side. By noon the peculiar concretion was much extended—the pulse was small and quick—the countenance depressed—the breath very fetid. Our author had no muriatic acid at hand, and therefore he mixed up some finely powdered alum, with just as much fluid as was sufficient to form a kind of paste, which he applied to the tonsils and parts affected, by means of the handle of a spoon. In the evening the fetor of the breath was much diminished. Another application of the alum was made; and by the next morning the tonsils were beginning to clean—the glands to subside. Three more applications of the alum completed the cure.

In the same village he saw a child of three years of age, who shewed symptoms of incipient disease of the throat. Dr. B. warned the health-officer of the impending danger, but the latter laughed at him, and said he cured all these throat-affections easily by acid gargles. Forty-eight hours afterwards the breath became fetid—croupal symptoms came on, and the child died suffocated on the following day. Another child soon experienced the same fate, there being only time for one application of the alum before death. A third was taken more early, and by the assiduous application of the alum, life was saved.

On the 15th of July, Dr. B. saw the brother of the child first-mentioned, aged four years. His pharynx was covered with concretions, which extended beyond the view—the cough was croupy—and he had, in Dr. B's presence, an attack of croupal suffocation, leaving no doubt in our author's mind, that the diphtheric inflammation had extended deeply into the air-tubes. An emetic had been given, and blisters had been applied to the nucha and legs. The mercurial treatment was proposed, as the only resource, but not assented to by the parents. The little patient sunk next day.

At this period the infant that had been weaned, when the mother became ill, was taken with the angina maligna; and the aluminous application was not put to the trial till the third day of the disease. It was then used, and some calomel given. This child was saved, though with much difficulty. Several other cases are related—some successful, but others not so. As far, however, as the limited number of instances, in which the remedy was employed, can afford evidence, the alum appears to be a valuable local application in this dangerous disease.

---

### 6. ON GREEN TEA.

Mr. Newnham has recently published a little brochure on this beverage, so refreshing and exhilarating to some—so distressing, we might almost say, poisonous, to others. This difference of effect, from the infusion of green tea, is attributed by our author, to a difference in the state of the system at the time the beverage is taken. Mr. N. is led to conclude that green tea is a direct sedative, in those head-aches, "*in which there is an increased action of its vessels, with more or less of power to support it;—or in other words, in a state of sthenic excitement of the organ.*" Thus, green tea is found to be particularly useful in the head-ache produced by the stimulation of alcoholic fluids; and in a similar affection arising from intense mental exertion or literary research. In such circumstances, it soothes irritation—"calms the nervous system—invigorates the animal frame—refreshes the jaded spirits—clears the ideas—brightens the faculties," and recruits the flagging energies of the brain. Such is the flattering picture of green tea, as drawn by Mr. Newnham. But then this picture has two sides. If the plant, in question, produces such delightful effects in *sthenic* excitement of the brain, or in increased vascular energy; it is to be remembered that, in opposite conditions of this organ of the mind, green tea is baleful. Thus, females endued with a high degree of *mobility* of the nervous system, existing independently of any *sthenic* excitement—and males, in whom the *asthenic* diathesis prevails, are they who generally complain of the effects of green tea.

The author has made many experiments on himself and others, with the view of ascertaining the above points, and if the histories are accurate (of which we have no doubt) his conclusions are just. One or two instances we shall glance at rapidly.

1. Mrs. Wicher was thrown from a gig on the 15th October, and suffered a *concussion of the brain* among other serious injuries. She had remained in a state of insensibility for 36 hours, after which, delirium alternated with transient gleams of reason. On the 18th, our author found her delirious, pulse hard and contracted, tongue furred and dry, skin hot, bowels torpid. She had great pain in her head and intolerance of light, with contracted pupil, injected conjunctiva, and disposition to drowsiness. She was largely bled, freely purged, and her head well leeched. 19th, The symptoms being very little relieved, the depletory means were again employed. 20th, The delirium had increased, and the head-ache was still intense. "She was altogether in a precarious state." She was now directed to take some strong green tea, with the view of arresting the inordinate action of the cerebral vessels, which still continued. The effect was remarkable. The pulse became fluttering—palpitation and sense of faintness succeeded—a cold moisture broke out on the skin—and, from a constant muttering, and confusion of ideas, she became calm, and remarkably collected. She sent for her husband, and said she felt herself dying—took leave of her children and friends—and then fell into a gentle sleep. After this she gradually and progressively recovered.

2. In the acute irritation of the membranes of the brain, in children, the efficacy of green tea has been strongly marked, in Mr. Newnham's practice. Exhibited during the early symptoms, as soon as a sufficient quantity of blood has been drawn, "and before the primary irritation has terminated in serous effusion, it has proved a more powerful means than any other we possess, of controlling that morbid action, which, if suffered to proceed to its second stage, is scarcely to be overtaken by treatment."

3. Mr. N. thinks that green tea promises to be very serviceable in the commencement of the cerebral disturbance attendant upon the continued fever of this country; and indeed so long as that excitation is continued, without any marked loss of power to support it. He has tested its effects in these cases, and speaks in confident terms of the utility of the beverage. "Thus, it has often produced quiet sleep, when great vigilance had preceded, and where opium could not, with propriety, have been exhibited. It has allayed the irritability of excitement, and *refreshing slumber* has been the consequence."

4. The next case related is that of the author himself, of which we shall present our readers with a short account.

In the spring and summer of 1822, Mr. N. was attacked with increased arterial action of the cerebral vessels, for which he was repeatedly bled, generally or locally, with strict adherence to the antiphlogistic system. But by these means the head-ache and throbbing were only palliated, not dissipated. He determined to try green tea. The pain was very intense, when he first employed the remedy, and the effects were memorable. Soon after swallowing a strong infusion (one ounce of gun-powder tea to the pint of boiling water) the severity of the head-ache was diminished, and a delightful calm stole upon him, accompanied by a considerable exhilaration of spirits. But this Elysian tranquillity was soon changed for a variety of painful sensations—an almost insupportable præcordial anxiety—palpitation and fluttering of the heart—general tremor, and a peculiar distress which he has no words to describe. Yet his spirits did not forsake him in this trial; for he felt that his head was better, and was rejoiced to know that he was in possession of a mean of mitigating his complaint.

His friends, however, were alarmed for his safety. The symptoms of agitation gradually subsided, and he slept that night with greater quietude than usual. During the progress of his illness, he had daily recourse to the remedy, for some time, and always with the effect of quieting the excitement of the cerebral vessels—accompanied, however, by the same train of nervous symptoms as above described.

Since his recovery, he has tried the green tea on himself and some of his pupils in health. The effects corresponded with those already described, and the general conclusion is, that green tea is a sedative—and that it will prove highly serviceable in all cases of high arterial excitement. It is abundantly evident, however, that this witching beverage is no sedative for *nervous irritability*. On the contrary, in such constitutions, and under such circumstances, it is extremely improper—we had almost said dangerous. We hope the remedy will be found, in other hands, to produce the effects described by Mr. Newnham. If so, green tea will be an addition of some value to the materia medica.

---

### 7. SEVERE HÆMATURIA UNEXPECTEDLY CURED.

The following case, by Dr. Chastaingt, is worthy of perusal. M. T. had been subject, for several years, to a hæmorrhoidal discharge, which, at one period, was very profuse. This discharge gradually ceased, and then came on a hæmaturia, preceded by certain constitutional symptoms, as cephalalgia, malaise, lassitude, faintness, weight in the loins, uneasiness in the bladder, &c. These symptoms gradually abated as the hæmaturia became copious; and they were renewed before each hæmorrhagic effort afterwards. The blood was sometimes intimately mixed with the urine—sometimes it came away pure and separately. In general, the exercise of walking or riding, as also stimulating diet or medicine, augmented the discharge. The patient experienced no difficulty or pain in making water; nor at the neck of the bladder or extremity of the urethra. The catheter had been introduced, but no material obstruction existed. Our author concluded that the blood came from the capillaries of the bladder, or from varicose vessels about the cervix of that organ.

Low diet, repose, cold applications to the hypogastrium and perineum, cool and acidulated drinks, emollient, and afterwards astringent injections into the bladder, together with the other usual means employed in hæmorrhagic complaints, were here used, without the least success. The patient's strength daily decreased, and a constitution previously robust, appeared to be fast breaking up. It was at this period that Dr. C. was called to the case, and he continued similar treatment for some time, without making the least impression on the disease. In fact, the more blood that was taken from other parts of the body, the more copious became the hæmaturia. Tonics, astringents,

&c. were next tried, without any beneficial result. A memoir of the case was then carefully drawn up, and transmitted to Paris, for the advice of the Metropolitan Lions. One of the most distinguished surgeons gave it as his opinion that the blood was furnished by the capillaries of the bladder, and that the treatment which had been adopted, was the only one which was proper; but he thought depletion was carried too far, and to that he attributed the want of success. Dr. C. at this time determined to examine the state of the bladder himself, and therefore introduced a sound into that organ. At the neck of the bladder, it met with some resistance; but this being overcome, nothing could be detected in the hollow of the organ. Dr. C. now imagined that the resistance to the sound might have been caused by some varicose veins, and that these might also have been the seat of the hæmorrhage. He therefore allowed the sound to remain in the bladder, in order to exert a salutary pressure on the supposed dilated vessels. The presence of the sound occasioned acute pain, and by the succeeding day, a sharp inflammation of the urethra (urethrite) obliged him to remove the instrument. Then, for the first time, the patient made water spontaneously unmixed with blood. Leeches were employed to remove the urethral inflammation, and there was no more hæmaturia. Eight months have since passed away, without any return of the disease.—*Biblioth. Med. Dec.* 1826.

Without attaching much importance to the author's opinion respecting the precise nature of the obstruction at the neck of the bladder, the case is worthy of record, as affording a hint on a similar occasion. It is in this way, indeed, that solitary cases become beneficial in practice, even when originally published with the view of supporting some false or favourite position.

---

### 8. CHOREA, FATAL.

A fatal case of chorea is so rare, that the dissection of a choreal patient is a treat to the pathologist. The following case occurred in the hospital practice of Dr. Hawkins, and is detailed in the March Number of the Medical and Physical Journal.

*Case.* Eliz. Smith, aged 17, was admitted into the Middlesex, on the 5th September, 1826, having suffered, seven weeks previously, from a severe attack of rheumatism, chiefly in the knees and shoulders. This had got better, and a fortnight before the date of her reception, she had been seized with involuntary convulsive motions in the legs, arms, and neck. These had continued ever since, with great violence. The catamenia had been suppressed for four months. She had now complained of headache, thirst, and pain in her back—pulse 96—tongue loaded—bowels constipated. Dr. Hamilton's plan of purgation was fully but unsuccessfully employed. The calomel, senna, turpentine, &c. never failed to dislodge copious and dark-coloured motions; "but they procured not the smallest alleviation of the convulsive spasms, which resembled those with which hydrophobia exhausts its victim." The unfortunate patient could not hold her head quiet for a single minute, and the grinding of the teeth was so violent as at last to force out the incisor teeth from the lower jaw. The convulsions were uninterrupted, except by short intervals of broken sleep. The intellectual faculties did not seem to be impaired. On the second night after admission, she was ordered into a warm bath, which measure, unfortunately, aggravated the convulsions, "and produced such an accession of irritation, and even of inflammatory symptoms that it was deemed necessary to take from her sixteen ounces of blood." The blood was inflamed. The bleeding was repeated on the following day, but produced very little alleviation of the spasms. After the failure of the purgative plan, a short trial was given to musk, without any effect. Camphor and opium succeeded in procuring some sleep; but after the second administration of these remedies, she slept soundly—awoke, and soon afterwards expired, 13th of September.

*Dissection.* No morbid appearance could be discovered within the cranium. In the upper part of the lungs there were some tubercles of a large size, and several earthy concretions were deposited in various parts. There were some adhesions between the liver and surrounding parts—"intestines healthy in appearance"—omentum and mesentery studded with numerous cysts, some containing a black semi-fluid matter, others containing calcareous depositions. Several large concretions were also found in the substance of the pancreas. The uterus was rather large and vascular, and the lining membrane of its body and fundus highly injected. The fallopian tubes and ovaries contained a good deal of the black matter above-mentioned.

Dr. Hawkins observes, that, granting irritation of the brain and nervous system to be the proximate cause of chorea—"sufficient cause for such irritation was met with in the preceding case"—viz. the earthy concretions. We cannot accord in this opinion. Surely these concretions were not the product of three weeks, the date at which the convulsions began—and if they previously existed, which they certainly did, why did they not produce irritation? It is evident, indeed, that the dissection threw no light on the cause of chorea, and therefore we must still regard it as capable of being produced by irritation which leaves no cognizable trace of its existence in the dead body. Dr. H. does not say whether the mucous membrane of the stomach and bowels was carefully examined. From the state of the secretions, and excretions, it is manifest that the digestive organs were in a very deranged state, and it would have been very desirable to have minutely investigated the whole internal surface of these organs; for *there*, we think, the source of irritation existed. From the numerous depôts of melanoid matters, in this case, we apprehend that this unfortunate girl would have ended her days under that terrible malady, *melanosis*, even had she escaped from the convulsions. Finally, we question whether this disease was fairly entitled to the name of CHOREA. Was it not more properly CONVULSIONS?

### 9. ERYSIPELAS—CONTAGIOUS.

In the March No. of the Medical and Physical Journal, Mr. James Arnott has published a paper, and related some cases of erysipelas, tending to shew the connexion of this complaint with affection of the throat, and also affording presumptive proof of the occasionally contagious character of the disease. Mr. A. has also insisted on the propriety of limiting the term "erysipelas" to the phlogosis, as it affects the face only, and applying the designation of cutaneous inflammation to those phlogoses spread over other parts of the body. For the reasons which Mr. A. assigns for this limitation, we must refer to the original paper:—the facts, or cases detailed, we shall here analyze. These cases occurred in one family—the mother being first seized with pharyngitis, terminating in mortification—then the husband was attacked with inflammation of the throat and erysipelas of the face—lastly the daughter, who had nursed the father, was affected with inflammation of the pharynx, and severe erysipelas.

*Case* 1. Mrs. M. aged 45, complained, on the 22d June, of pain and difficulty of swallowing, though no redness could be perceived internally, or swelling externally. No fever. *An emetic—calomel and James's powder—saline aperient.* 23d, Throat affection continues, but still no redness was visible—refers the pain to a spot behind the larynx—slight heat of skin, and frequency of pulse. *Leeches to the throat—saline antimonials.* 24th, Had a restless night, with distressing sense of dryness and constriction in the throat, whenever she dropped off to sleep. Pain of deglutition increased, and a troublesome expuition of phlegm—some tumefaction in the anterior part of the neck, with tension and tenderness—slight hoarseness—no cough—breathing free—pulse frequent, but resisting. Venesection to 18 ounces—leeches to the throat. The blood was cupped and buffed—tension of the neck abated—expresses herself much relieved—deglutition easier. This amelioration continued till the evening, when an exacerbation came on, with flushed face, frequent, but unresisting pulse. In consultation with Dr. Macleod, it was resolved to give an opiate. But symptoms of sinking soon came on, and early on the morning of the 25th, stimulants were exhibited. She rallied a little, but died next morning.

On *dissection*, the inflammation was found to be very limited, occupying that portion of the pharynx only which borders on the aperture of the larynx. The inflamed part did not exceed the size of a shilling, and exhibited a small spot of mortification, close to the rima glottidis, not larger than a silver penny. "The slough (for it was surrounded by a distinct line, as if Nature had begun the work of separation) occupied the right lip of the opening of the larynx, from the root of the epiglottis to the corresponding arytænoid cartilage, without, however, descending into the cavity of the larynx itself, which was free from disease."

*Case* 2. June 29th, Mr. M. the husband, who had been in constant communication with the deceased, had been complaining for two days of soreness in the throat. He has now painful deglutition, with diffused redness over the posterior fauces, without much swelling. 30th, The inflammation of the velum, tonsils, and posterior pharynx has increased, with a blush of redness and tumefaction on the upper eye-lid. 1st July, Erysipelas had developed itself on the eye-lid, and was extending to the cheek. We need not go farther into the details. The case was severe, the erysipelas having occupied the face, extending to the scalp, and requiring nearly a fortnight to make its circuit, and more than three weeks, for recovery. The affection of the throat continued for a few days, and then disappeared. The treatment was, at first, strictly antiphlogistic; and, when the violence of the disease had abated, stimulants and tonics were resorted to, with opiates at night.

*Case* 3. Miss M. aged 20 years, lived in the country, till the time of her mother's death, when she came to town, and nursed her father, as before-mentioned. When his skin was in a state of desquamation, she first complained of head-ache and sore throat. July 22d, Next day, the febrile symptoms were considerable, as were the pain and difficulty of swallowing. The velum, uvula, and tonsils were of a bright red colour, and the posterior surface of the pharynx was covered with a yellow glairy mucus, which could not be detached. 24th, Vesicles appeared on the nose, and erysipelatous redness occupied the left cheek, spreading thence to the rest of the face, and also to the hairy scalp, making the circuit of the head by the 2d of August. In both the latter cases, there was an irritable state of the bowels, with diarrhœa, for some time. The inflammatory action of the throat continued several days, and then disappeared. The antiphlogistic treatment, with the addition of one venesection, to 16 oz. was employed at the beginning, and ultimately tonics, but no wine.

For many interesting and sensible remarks on these cases, and on erysipelas generally, we must refer to the original communication. We have here only laid the facts before our readers. One inference, however, we think they will all draw—that the husband and daughter took the disease from the first patient.

---

### 10. THE PROTEIAN MALADY.*

The following singular case is drawn up and published by the patient, himself a medical man—M. Sarrut. The observations made in this way, are of high interest; for patients who are unacquainted with the structure and functions of parts, often give very vague and erroneous statements of their feelings and symptoms.

For five years, M. Sarrut had been afflicted with such violent palpitation of the heart, that he expected nothing but sudden death. At length, however, he became familiarized with this fearful symptom, and began to make observations, and to reason on his own case, from ex-

---

* Journal Complementaire.

perience rather than from the theories or speculations of authors. He dares not give his malady a name, but exhibits a faithful delineation of the phenomena, leaving the nomenclature to others.

M. S. was born of healthy parents, and enjoyed uninterrupted health himself, till the age of twenty, with the exception of some convulsive attacks in his early youth, when much excited mentally. At the age of 20, he was very tall and robust, but endued with a mind unusually ardent, and perhaps morbidly sensible. At the age of 21, this gentleman experienced some terrible moral affliction, the nature of which he does not state, nor is it necessary to be known. He exerted all his fortitude and philosophy to bear up against the evil—but all in vain! It bore him completely down, and he ceased to struggle with the *moral* affliction. But a *physical* affliction soon followed, which claimed its share of attention from the unhappy sufferer. One day, while reading a letter, he felt his limbs grow rigid—he was seized with a convulsion—the blood seemed to concentrate about the heart, which palpitated violently, and he was on the point of suffocation. Antispasmodics and stimulants, by restoring sensibility, only brought the victim to a sense of his own miseries, mental and corporeal. His appetite now entirely failed—he passed the nights without sleep, counting the pulsations of his heart—his mind was the seat of despair! The consolations of friends were of no avail, but rather aggravated the moral affliction—and therefore he indulged to the utmost, in mental despondency and anticipations of corporeal dissolution. Perhaps this was as good a course as any. Time softened down, and indeed annihilated the anguish of the *moral* evil—but the *physical effect* remained. The convulsive attack before alluded to, was followed by several others, without any antecedent moral cause or mental emotion. To check these attacks the patient had recourse to *narcotics*, in the autumn of 1822, twenty months after the invasion of the malady; for then antispasmodics had entirely failed to produce any effect. He also kept to a very restricted diet, avoided coffee, liqueurs, wines, strong animal food, and *fish*, which last article of diet always brought on the palpitation. Constipation was obstinate. The patient was forced to apply from 25 to 40 leeches once a month to the region of the heart. For a fortnight after each application, the *pulsations* were equally frequent as before, but the *palpitation* was not so violent. During the above year (1822) the heart seldom beat less than 120 to 140 in the minute. There was constant pain in the region of the heart, and, as nearly as M. S. can conjecture, in the left ventricle of that organ. His daily avocations (he observes) dissipate, in some degree, or at least render him less sensible of this pain; but it is particularly increased when he attempts to write. The pulsations of the heart sometimes cease all at once, and then the patient experiences an indescribable feeling of weight or anxiety in the præcordial region, with sense of suffocation, and vertigo. This state lasts two or three minutes, during which, the patient is scarcely sensible enough to prevent his falling to the ground.

After emerging from this state of stupor, M. Sarrut experiences an extreme appetence for warm fluids. At these periods, he often drinks water so hot that he can scarcely hold the cup in his hand, or bear the touch of the water or cup on his lips. The hotter the water, the greater solace he finds from the beverage. He generally drinks three or four bowls of hot fluid on getting out of bed, the same on going to rest—and a large quantity after each attack of the kind described above. He lives in the country—is fond of horse-exercise, and it generally agrees with him.

The year 1823 passed away in the same melancholy condition. No appetite—no good sleep in bed—the greater portion of the nights is spent in sitting up, or lying on a carpet on the floor. The emaciation is considerable, but yet he has a good deal of muscular strength, when he exerts it. Towards the close of the year, M. S. made several journeys, and found himself sensibly relieved by these tours. In the course of the winter of 1824, the attacks above-mentioned ceased, and the patient had become so habituated to the palpitation that he did not pay much attention to it, though he still adhered to a restricted regimen. He found also that he was able to withstand, without much emotion, some moral afflictions that, two years previously, would probably have destroyed his life. Gradually, his fortitude of mind and health of body increased, till he might be said to be quite recovered, except that the least over-exertion of body, or any excitement of mind renewed the palpitation. After nearly a year of tolerable health, M. Sarrut experienced suddenly, and without any ostensible cause, on the 30th May, 1825, a prolonged dimness of sight, vertigo, and sense of bad taste in the mouth. His skin felt dry—he went to bed—and drank freely of warm tea. At the end of 24 hours, his skin was still arid, his tongue furred, his head painful, his taste depraved, the bowels affected with colicky pains, and he had a sense of deep-seated uneasiness directly under the sternum. All this time the heart was perfectly tranquil. He had made no water, nor had his bowels acted during these 24 hours. Lavements failed to open the bowels, and at the end of 30 hours, he made some high-coloured urine in small quantity. The abdomen was tense, and his skin dry. He drank large quantities of diluents. At the end of 44 hours the bowels were still confined, and the skin arid; but the nervous system was quiet. He took two grains of emetic tartar, which caused efforts to vomit, but no discharge from the stomach. Violent colicky pains and head-ache now succeeded, and copious stools were procured. He fell into a sleep which lasted four hours, on awaking from which, the urine flowed, and the skin was moist, but the old palpitation had returned. No appetite—rigid regimen continued. At the end of the fourth day, he experienced severe pain in the region of the heart, accompanied by evident febrile action in the system, intense head-ache, extreme heat, sense of suffocation, palpitation, delirium. This state continued for the space of seventeen hours, with urgent thirst for hot fluids. During the next fortnight he had a dozen of these attacks, but without any regularity as to the hour of their accession. He had now a total disgust for food, with almost constant perspirations, and heat of skin. During the attacks, he was threatened with imminent suffocation, and the pain in the region of the heart was excruciating.' It appeared as if a bolt of red hot iron was driven through the heart! His voice became extinct, he emaciated rapidly, and his bodily powers were in a complete state of prostration. His bowels never acted without assistance—his urine was very scanty—his legs became œdematous—he was enfeebled to the last degree—but his mental faculties were unaffected. On the 15th day, all these symptoms had arrived at the greatest degree of intensity. Cold vinegar and water was applied to the head—sinapisms to the feet. A diarrhœa came on, and some feeling of appetite returned. On the 20th day he was able to take some soup and bouillie for the first time. He went on better till the 28th of June, but still suffered severely from the pain in the region of the heart. On this day, during a storm, he was seized with violent head-ache, and dreadful palpitations, which could be plainly heard by the bye-standers, for several hours. During the month of July he had many attacks of the kind described. He was now extremely emaciated, and his mental faculties suffered occasional aberrations of short duration. His memory failed, and the action of the heart was often as quick as 180 pulsations in the minute. The palpitation could be plainly heard at ten paces distance from the patient. On the 27th July, he had an unusually severe paroxysm, and the sense of suffocation was most urgent. The countenance assumed a yellowish coppery hue, the extremities were cold, and the patient could hardly turn himself in bed, so little muscular power did he possess. In the evening, the palpitation ceased, but the heart seemed ready to burst. The pulsations were now only 30 in the minute, and an icy coldness spread over the body, accompanied by cold sweats. At eleven o'clock, the head-ache was insupportable, the face livid, the breathing stertorous, and the ideas incongruous. Fifteen leeches were applied to the temples, and the cephalalgia was relieved; but there was great embarrassment of speech and respiration. Sinapisms were applied to the feet, and the afflicted patient sunk into a sleep, or rather a *cessation of sensibility*, for a quarter of an hour, from which he was roused by a most acute pain in the region of the heart, and a vivid lucidity of ideas. The blood now burst from the leech-bites on the temples, and flowed in great abundance. But the sensations of icy coldness, especially in the chest, were terrible, and the patient thought himself on the very point of death. At this time sinapisms were applied to the chest and abdomen, with cupping-glasses below the clavicles. He now once more sunk into a tranquil sleep, which lasted till five o'clock in the morning. When he awoke, he was deluged with blood, which had burst from the nose. It was calculated that he lost, in this way, more than 40 ounces. He also threw up blood by vomiting, and the debility was extreme. The nasal hæmorrhage and hæmatemesis went on alternately through the forenoon, and at mid-day he fell into a tranquil sleep, from which he awoke with no other disagreeable sensation than that resulting from the sinapisms. From this time, he went on so well, that, by the 11th of August, he was able to travel in his cabriolet. On the 24th, he undertook a journey of 200 leagues to the Pyrennees. After some stay there, he returned to his family, near Toulouse, where his health was completely re-established, and continues so to this day. He has adhered, however, to the most rigid and systematic regimen, during and subsequent to his illness.

Several of the most eminent physicians of the French metropolis pronounced the disease to be aneurism of the heart. It is very evident that it was no such thing. But what was the disease? In our humble opinion, it was neither more nor less than that Proteus called DYSPEPSIA. The terrible forms which it assumes—the horrible diseases which it imitates—the insupportable feelings which it induces—are not known to one in five hundred of the profession, even in this advanced æra of medical science! We could relate cases still more strange than the foregoing—cases that crowd upon our notice, and hourly excite our astonishment at the prevalence and domineering influence of this portentous malady. But more of this hereafter.

### II. ON LUNAR CAUSTIC.

In preceding numbers of this Journal, we have noticed the observations and experiments of Mr. Higginbottom, of Nottingham, in respect to the external application of the nitrate of silver in various affections. In the April number of the Medical and Physical Journal, Mr. H. continues to favour his professional brethren with the results of his farther experience.

Mr. H. is very particular in directing the attention of practitioners to the proper mode of applying the caustic. He uses it in the solid form, first slightly moistening the surface to which it is to be applied, with pure water—except in the case of ulcers, from which lymph or pus exudes. If the caustic be passed *once* slightly over the moistened skin of any part (except the hand, where the cuticle is thick) an eschar simply is formed. If passed *twice or thrice*, some vesication will be added to the eschar:—if still *more frequently*, there will be vesication alone. In the first case, there will be no pain—in the second and last, there will be soreness proportionate to the degree of vesication. These observations should be kept steadily in view.

1. *Recent bruised Wounds of the Skin.* In these cases the caustic should be applied upon the wound, leaving no spot untouched—and also upon the surrounding skin to the extent of one-third of an inch, in such a manner as to induce an eschar, without vesication. Moisture is then to be removed from the wound by linen or lint, and the skin surrounding that to which the caustic is applied, is to be moistened, and covered with gold-beater's skin. The parts are to be kept cool—free from covering—and exposed to the air. By this procedure an adherent eschar is generally formed, and no farther application or attention is required, except in old people, whose skins are sometimes irritable. In these cases, some fluid will form upon the edges of the eschar requiring evacuation by a small puncture. If the eschar be accidentally removed, the caustic must be repeated as before.

2. *Small Ulcers.* Mr. H. thinks he has improved greatly upon the mode of application in these cases, since his first publication. The surrounding skin is first to be moistened, and the caustic applied lightly, (so as not to induce vesication) to the extent of half an inch round the ulcer. It is then to be applied over the ulcerated surface, more freely than to a recent wound. The whole is to be covered by gold-beater's skin. The application of the caustic round the ulcer subdues the inflammation of this part, and induces a firmer and more adherent eschar. On the succeeding day, the gold-beater's skin is to be removed, by moistening it with water—"a small smooth slit is to be made by means of a pen-knife, through the eschar, in its central part, and then a little pressure is to be made, so as to evacuate any fluid which may have been effused." The breach in the eschar is to be repaired by re-applying the caustic, and the whole is to be protected as before.

On the first and second days there is usually little fluid secreted—during the succeeding five or six days, rather more is formed, and the same means are to be repeated, until the eschar becomes completely adherent, which will be ascertained by the indentations on the surface of the eschar, usually about the tenth day. During the unadherent state of the eschar, an efficient purgative should be given every second or third day, and rest enjoined. The portions of eschar, as they separate, are to be carefully removed by sharp scissors.

3. *Punctured Wounds and Bites.* In *recent* punctures, all loose portions of skin closing the orifice are to be first removed—the puncture and surrounding skin are then to be moistened with water—the caustic is to be applied to the *former*, until some pain be experienced, and over the *latter* lightly, so as not to induce vesication. The caustic is then to be applied to the skin for an inch round the puncture—and even to a greater extent, if the swelling exceeds the space. The part is to be exposed to the air. These cases are generally adherent from the first application of the caustic. Sometimes, however, this is not the case, and the caustic is to be re-applied.

At a later period of punctured wounds, inflammation is generally present—the punctured orifice is nearly closed—and some pus has usually formed. A slight pressure is to be applied to evacuate the humour, and the caustic is then to be introduced into the puncture and on the surrounding inflamed skin, and even beyond that space. The parts are then to be exposed and allowed to dry. In this manner an adherent eschar is formed, and the inflammation subsides. If an abscess have formed, it must be freely opened, and the caustic is to be applied within the cavity, after which a poultice of bread and water.

4. *External Inflammation.* In this case, the part is to be washed with soap and water, and wiped dry. The inflamed surrounding skin is then to be moistened, and a long stick of caustic to be applied flat upon the moistened surfaces, taking care that every part of the inflamed skin be touched, and also a circle of skin beyond the boundary of the inflammation. The caustic should be passed twice or thrice only over the said surfaces, and then exposed to the air to dry and be kept cool.

In twenty-four hours, the inflammation will have greatly subsided, or be checked, and at this period there is usually some vesication, which, however, never increases the inflammation or irritation. On the third day there is generally more vesication—the part has a puffy feeling, and is quite free from inflammation.

In cases of erysipelas from wounds or ulcers, the wound or ulcer and the inflamed surface are to be treated by combining the above modes of using the caustic.

5. *In constitutional Erysipelas*, bleeding, purging, and emetics are to be premised, and then the caustic is to be applied in the following manner:—viz. over the whole inflamed surface, and beyond it to a far greater extent than in phlegmon—to two inches on the sound skin. Any fresh accession of erysipelas is to be healed in the same manner immediately. Mr. H. believes that, by means of the caustic we have a complete control over the disease. If the erysipelas be attended with vesication, the vesicles should be broken, and the parts touched with the caustic. When the disease affects the head, the scalp should be shaved, that there may be no impediment to the application of the caustic.

6. *Phagedenic Ulcers.* The caustic is to be lightly applied to the whole ulcer, but particularly to its edges and over the surrounding skin. If situated on the glans penis, a little lint is to be left upon it—if on any other part, the cold poultice and lotion are to be applied.

Having no experience in this mode of managing inflammations and ulcers, we should deem it impertinent to make any remarks. We have laid the directions of Mr. Higginbottom fairly before the profession, and the remedy will doubtless be soon put to the test of farther experience.

---

### 12. CHRONIC DIARRHŒA.

There are many forms of this disease evidently dependent on ulceration or other organic lesion of the mucous membrane of the bowels, the consequence, or at least the sequence of dysentery. But there are some other cases of obstinate diarrhœa, where the disease goes on for years, and where dissection, after all, detects no organic change in the intestines. Dr. Baillie has described "a particular species of purging," which is but little known, and has generally proved fatal. The alvine discharges resemble a mixture of lime and water, with froth on the surface. It most commonly occurs in people who have resided in warm climates, and suffered from hepatic affections: but not exclusively in this class. When the disease is in a mild form, the evacuations are of the consistence of pudding, and of a pale colour. Under such circumstances, and especially if the motions be occasionally figured, the patients may live many years with the complaint. They have usually a sallow countenance—are thin, but not greatly emaciated—have tolerable appetites—white coated tongues. Nothing particular can be detected when the abdomen is examined by the hand. There is no tumour—no pain on pressure—but the bowels are generally distended with air. Dr. Baillie never had an opportunity of examining any patients who died of this disease, and therefore could not speak as to its pathology. But Mr. Wardrop, in a note to his edition of Dr. Baillie's works, informs us that he (Mr. W.) had an opportunity of dissecting a patient who had been under Dr. B's care for this complaint, and that he found considerable thickening of the coats of the rectum and colon, great contraction of the calibre of the gut, with small, but deep ulcers interspersed over its surface. Dr. Seymour and Mr. Arnott, however, have each had an opportunity of examining the intestinal canal in this complaint; but in these instances, there was no breach of structure or organic alteration of any kind in the large or small intestines.

We have been induced to notice this subject in consequence of a remedy which has been introduced of late by Dr. Elliotson, at St. Thomas's Hospital—namely, the sulphate of copper, combined with opium. This zealous physician has given the remedy in a considerable number of cases of chronic diarrhœa, where all, or almost all other remedies had failed, and with complete success, in every instance. The dose is generally half a grain twice a day, with half or a grain of opium, increasing the dose to two or three grains in the day, but seldom beyond that quantity. We understand that Dr. E. made experiments with the opium alone, which failed to cure the patients—and the reason why he combined it with the sulphate of copper, was to prevent the latter from causing pain in the stomach and bowels.

Dr. E. is inclined to view the remedy in respect to its modus operandi, as simply an astringent; but when we reflect on the power which this sulphate possesses of allaying irritability when applied to external sores, we shall be induced to attribute much of its success in these cases, to its action as lessening morbid irritability of the intestinal canal. But as Dr. Elliotson's observations will probably soon be published, we shall defer any farther remarks till that period.

### 13. ANEURISMAL TUMOUR, WITH UNUSUAL SYMPTOMS.*

Captain Fermin (naval) of active and decided character, had had venereal ulcers at the age of 20, which were cured without mercury. At 25 he contracted, for the second time, a gonorrhœa which, after a debauch, suddenly disappeared, and was succeeded by pain and tenderness in the epigastrium, with periodical cramps and pains in his stomach, kept up by irregular living and food of difficult digestion. Emetics and purgatives, which were administered, rather exasperated the complaint. After this, he had yellow fever in the West Indies—received several wounds in different naval actions—fracture of four ribs—

* Professor Lallemand, of Montpellier. Repertoire, No. 4.

experienced shipwreck thrice—and, to crown all, was three years a prisoner in the Pontons in England! Such were a few of the events of this officer's boisterous passage through life. At the age of 43, he experienced wandering pains in various joints, generally with a diminution of uneasiness in the stomach. The pains settled severely on one knee, and required leeches for its removal. At the end of a year he had another attack of the pain in the knee, with corresponding relief to the stomach complaint. Fifty leeches, fomentations, and other measures failed, this time, to remove the pain of the knee. In the course of three months a succession of blisters round the joint exasperated the complaint. The patient, who was a most observant man, felt a pulsation within the joint, which his medical attendants paid no attention to, considering the disease to be rheumatism or gout. Another set of attendants suspected syphilis, and employed mercury, with evident disadvantage. The knee-joint increased rapidly in size, and the pain was augmented. The surgeons now acknowledged that there was a pulsation in the joint, but they were not agreed as to its nature or cause, nor the means to be used as remedies. The patient, after seven months of suffering and uncertainty, came to Montpellier, (March, 1826) for the purpose of having the limb removed. But he consulted an eminent surgeon of Toulouse, on his way to Montpellier, who gave it as his opinion that there were indications of two aneurismal tumours; but that it was difficult to decide whether the pulsations depended on a real dilatation of the articular arteries,—on a fungus hæmatodes—or on some other disease of the knee-joint. Possessed of this opinion, the patient presented himself before M. Lallemand, with the following phenomena. His age was 45, but he had the appearance of being at least 60 years. His complexion was pale and yellowish, and his countenance indicated great sufferings. The right lower extremity seemed wasted, especially below the knee, which last was nearly twice its natural size, surrounded with varicose veins, and the skin tense and shining. The leg was bent on the thigh—all voluntary movements nearly obliterated; and when any motion was communicated to the joint, there was considerable pain—head of the fibula very prominent—excessive pain extending from the head of this bone, and following the course of the peroneal nerve. When the patient held in his breath, the veins of the knee became doubly dilated, and the skin blue. The same phenomena occurred when the limb was pendant; but when raised above the level of the body, the knee regained its natural colour, and the dilated veins disappeared. The upper extremity of the tibia was about double its proper size; and at the inner side of the ligamentum patellæ was an oblong flattened tumour, resembling the half of an egg, communicating a distinct sense of pulsation, synchronous with the action of the heart, and accompanied by a feeling of expansion at each stroke. On the external side of the patella, in front of the head of the fibula, there was another tumour prominent beneath the skin, but very mnch smaller than the first, presenting, however, the same pulsatory movements as its neighbour. The pulsations in both could be completely stopped by pressure on the crural artery.

M. Lallemand was at first inclined to think there was an aneurism of each inferior articular artery: but, after observing that the ligamentum patellæ itself was bulged out—that, on making firm pressure with the finger on one of the small tumours, a deep indentation could be formed, at the bottom of which, M. Lallemand thought he could feel a circular aperture leading into the main swelling—that on compressing either of the small tumours, the other was rendered more prominent—our author came to the conclusion that there was an aneurismal tumour developed in the interior of the bone, (tibia) which had become enlarged and extenuated—that the ligamentum patellæ, by offering a greater resistance than the parts on each side, had caused the appearance of the two neighbouring tumours—and that the saliency of the head of the fibula was the result of enlargement in the head of the tibia.

Ligature of the crural artery now presented itself as the best remedy, and the operation was joyfully acceded to by the patient. Messrs. Dubreuil, Dugés, and Dunal were called into consultation, and concurred in the propriety of the measure. The artery was isolated and taken up in the middle of the thigh, but its parietes were observed to be unusually thickened. The patient had borne the operation so far, without a single complaint; but when the ligature was drawn, he uttered a piercing cry, and observed that he felt a burning pain in the direction of the artery. As M. Lallemand was certain that he had not included the nerve, he drew the other knot, without taking any notice of the circumstance. One end of the ligature was cut away, and the wound closed. The tumours were lessened in size, and all pulsation ceased. As the patient still complained much of the pain in the line of the vessel tied, M. Lallemand was anxious to prevent arteritis, and therefore abstracted a pint of blood from the arm, which instantly relieved the pain. The venesection, however, was repeated in the evening, with the best effect. The wound closed by the first intention, and the limb was less painful, and more capable of spontaneous movements. On the 8th day after the operation, the heat of the knee was above that of the other, the movements more free, the swellings diminished. On the 15th day, the motion of the joint might be said to be entirely returned. The tumours, however, receded very gradually, as did that of the head of the tibia. In two months the patient got out of his bed. On hanging down the limb, the knee still got red and swelled, and pressure on the head of the fibula caused pain in the direction of the peroneal nerve. Ice applications dispersed these last symptoms. In three months, the patient could walk without crutches, and he went to Bagneres, where the baths accelerated his recovery*.

* In a letter at the end of the Journal, Professor Lallemand informs the Editors, that the patient is perfectly recovered—can walk without crutches, and is freed from the stomach complaint under which he had so long laboured, by the regimen enjoined by the Professor.

### 14. M. BRESCHET ON SANGUINEOUS TUMOURS OF EQUIVOCAL CHARACTER.*

The foregoing case of Professor Lallemand has induced M. Breschet to investigate the subject farther; and the results of his researches and observations are given in a paper of considerable length, in the same journal. We shall endeavour to condense the communication into as small bounds as are consistent with perspicuity, for the benefit of our readers.

M. Breschet divides the inquiry into four heads;—1mo, Has this affection (M. Lallemand's case) been described by surgical writers?

2ndo. Is the disease an aneurism?

3tio. Can it be confounded with other affections?

4to. Is ligature of the principal vessel the best method of treatment; and should it be practised in all cases?

Fabricius Hildanus records a case which bears on the present subject. A young woman had a tumour at the upper part of the leg, which was swelled to the size of a person's thigh. The limb was covered with ulcers and the head of the tibia as if by a development of its cellular structure. The young woman died, after a tedious illness. Ruysch has also reported some cases of the kind, but not with sufficient details. M. Breschet then proceeds to English authorities, and extracts several cases from Pott, Pearson, and others, which resemble much the case by M. Lallemand, though they were not considered at that time to be of an aneurismal nature. We shall therefore pass these over, and come to recent cases taken from the practice of M. Dupuytren, in the Hotel Dieu.

*Case* 2. Clement Renard, aged 39 years, reported that seven years previous to his entrance into hospital, a tumour appeared at the upper and inner part of the right tibia, just below the knee-joint, which he said communicated pulsation to the hand. On the 9th February, 1819, when received, the tumour was not large; the skin was red and thin; there was a distinct pulsation perceptible in it, synchronous with the action of the heart, which pulsation ceased when the crural artery was compressed. M. Dupuytren was of opinion that the tumour was caused by dilated arterial capillaries, and that there were probably organic changes taking place in the soft parts and also in the bone. Cold washes were applied, and pressure was made on the line of the artery in the thigh, but without any benefit. The surgeon then determined to tie the artery, which was done on the 16th March, 1820. The ligature caused no pain when drawn, and the pulsation of the tumour ceased. Next day the tumour was diminished, and communicated no pulsation —the limb preserved its sensibility and flexibility. All went on well till the 14th day, when there was some hæmorrhage from the wound. On the 16th day (the ligature having come away) some pulsation was felt in the tumour, and that night another hæmorrhage took place. After this there was pressure maintained on the line of the artery for

* Repertoire, No. 4.

some time, and the patient was discharged on the 30th of April, cured. There was still a little swelling in the site of the original pulsating tumour, but no pulsation remained. A long time after this, the tumour regained its original size, and he returned into hospital on the 1st August, 1826. The tumour now extended from the knee one third down the leg, on the inside—the cutaneous veins were dilated—the integuments thin—no pulsation perceptible. The swelling measured 32 inches in circumference. M. Dupuytren amputated on the 5th of the same month. The man recovered and was discharged cured. The tumour, which is described by M. Breschet with great minuteness, was formed principally by an enormous enlargement of the head of the tibia. This osseous tumour was divided into several cells, some of which contained a gellatinous substance, and others coagulated blood, such as is seen in old aneurisms. The articular arteries were greatly dilated, as also the veins; but there was no aneurismal sac, unless the bony parietes of the tumour could be considered as such.

*Case* 3. J. Thevenim, aged 22 years, had strained the knee-joint twice. After the last accident, the joint swelled considerably, and was treated by leeches and the usual means. He entered the Hotel Dieu in March 1826, with a tumour situated on the external part of the right knee-joint, the size of one's fist, communicating a distinct pulsation to the hand, which pulsation ceased on pressure being made on the crural artery. M. Dupuytren pronounced the disease to be a sanguineous tumour of the upper extremity of the tibia, with degeneration of structure. He thought ligature of the artery would be useless, and preferred amputation, which the patient refused to submit to, and left the hospital. He returned again on the 3d May, in a still worse state, and M. Dupuytren amputated the thigh on the 5th of the same month. It was necessary to apply fifteen ligatures. Hæmorrhage took place two days afterwards—bad symptoms supervened—and he died on the 9th, four days after the operation.

On dissection the stump was found in a diseased, or at least unhealthy state, but no visceral lesion could be detected.

The tumour, on examination, was observed to be smaller than before the operation. When injection was thrown into the popliteal artery, it issued by a multitude of points from the internal surface of the tumour, which had been perpendicularly split. The tumour was like a honey-comb, and the various cells were filled with blood in concentric layers, the exterior layers being much paler than the interior. The outer shell was bone and various degenerated structures, of a fibrous, fibro-cartilaginous, ligamentous, and carcinomatous appearance.

*Case* 4. G. Lamizel, aged 33 years, was received into the Hotel Dieu on the 5th July, 1825. Six months previously to this woman's entrance into hospital, she sprained her ankle violently, after which accident, her foot swelled, became red and painful. Leeches had been applied, and the usual means resorted to; but rest had not been preserved in the member. A few days after the accident, a small tumour appeared, according to the patient's account, of a pulsating character, which gradually increased in size for five months, and then continued stationary till she entered the Hotel Dieu. She had consulted many surgeons, most of whom considered the disease as aneurismal. When examined at the hospital, there was a tumour on the instep, about an inch in elevation, and of moderate extent, without heat, redness, or any alteration in the colour of the skin. Deep-seated, but distinct pulsation was perceptible in the tumour, which ceased when the anterior tibial artery was compressed. The patient complained of severe pain in the part, depriving her of sleep; but her general health seemed good. M. Dupuytren could not make up his mind as to the precise nature of the tumour. He brought her into the operation room, and having all things prepared for amputation, determined to puncture the tumour by way of exploration. M. Breschet expressed an opinion that the tumour was vascular, and that its principal seat was in the osseous structure. A bistoury was plunged into the centre of the swelling, and only a few drops of black blood issued, and not per saltum. The incision was enlarged, and a soft, reticulated bleeding, and fleshy tissue presented itself, not unlike the corpus cavernosum penis, or rather the substance of the placenta. M. Dupuytren then determined on partial amputation of the foot, which was performed according to the method of Chopart. She was discharged cured in six weeks from the time of the operation. On examination, it was found that the disease had invaded the first, second, and third metatarsal bones, which were reduced into a sort of bouillie, in the centre of which was an actual cavern or cavity, filled with grumous blood. The other minute alterations of structure we need not notice. Great numbers of small vessels entered into the diseased mass.

Passing over M. Breschet's observations on the symptoms, mode of development, and structure of these tumours, we need only say that this distinguished anatomist comes to the conclusion that they are of an *aneurismal character*—" du caractere anevrismal." He thinks they have been confounded by surgical writers with various other affections, as osteo-sarcoma, fungus hæmatodes, inflammation of the veins of bones, and of the bones themselves.

### The Ligature.

If it be true that the seat of this disease is in the arteries of the osseous tissue, and that these vessels present a veritable dilatation, or " arteriectasia," it is natural to conclude that ligature of the principal trunk of these vessels would be a likely means of remedying the disease. M. Breschet is of this opinion, and appeals to facts in its favour.

The sooner, however, the ligature is applied, the greater chance of success; for it is evident that the more the disorganization of the osseous tissue has advanced, the greater will be the difficulty of cure, even when the aneurismal character of the complaint is removed by the ligature. M. Breschet remarks that, although the ligature has not always cured the disease, yet in no case has the pulsation and other phenomena of aneurism re-appeared. When, however, the operation has been so long delayed, that considerable disorganization has taken place in the various structures of the part where the tumour is situated, amputation offers the only chance of success.

### 15. MAMMARY DISEASES.

A neat and methodical view of the diseases affecting the human mamma is given by Dr. Cummin, of Glasgow, in the Ed. Journal for April, of which we shall present a condensed analysis in this place.

The following is the list of maladies to which this organ is liable:—Mastodynia, or mammary neuralgia—mastitis, or inflammation—hypertrophy—atrophy—struma—serous cysts and hydatids—fibrous tumour or pancreatic sarcoma—adipose tumour—encephaloid tumour, or fungus hæmatodes—carcinoma, including scirrhus and open cancer.

1. *Mastodynia.* Morbid sensibility of the mamma, so as sometimes to be painful on exposure to cold, or the touch of bed-clothes; this distressing sensation extending over the whole breast, or both breasts, and even to the shoulder and arm. In the simplest form there is no perceptible change in the part—on other occasions, one of the lobes of the mamma becomes slightly swollen, and then constitutes the "*irritable tumour of the mamma*," of Sir Astley Cooper. Justamond has treated largely on this complaint.

2. *Mammary Inflammation.* This is too common to need description. The phlogosis generally ends in abscess. Chronic inflammation of the mamma is of less frequent occurrence; but it is a disease of great importance, on account of its similitude to scirrhus. "This disease is distinguished by a hard, painful tumour, deeply seated in the mamma, accompanied with a feeling of heat, throbbing, and sometimes with darting pains through the centre of the tumour." If leeches, discutient applications, and a proper regimen are not employed, suppuration ensues, and the skin over the tumour becomes red, tense, and painful.

3. *Hypertrophy.* This is a morbid enlargement of the mammary substance, sometimes to a monstrous size. In the early stages, there are sometimes symptoms of local inflammation and constitutional disturbance. The organ sometimes acquires such a degree of magnitude as to require extirpation.

4. *Atrophy.* This absorption of the mamma usually takes place in advanced life, commencing after the cessation of the catamenia. It has also followed the use of iodine.

5. *Mammary Struma.* This, in its earlier stages, is not always easily distinguishable from diseases of a much more formidable character. Sometimes a hard lump forms in the mamma, and remains nearly quiescent for years: at other times, the whole gland is affected with scrofulous enlargement. In all cases there is a strong tendency to suppuration, and the matter is mingled with curdy flakes, forming the principal diagnostic of scrofula. In strumous disease there is always an enlargement of the mamma, instead of that contraction which occurs in one form of carcinoma. The tumour is tender when grasped—never possesses the stony hardness of cancer—and there is never retraction of the nipple.

6. *Serous Cysts and Globular Hydatids.* These have been found in the adipose substance of the mamma. They are not dangerous of themselves; but Dr. C. thinks there is reason to believe that they occasionally give rise to other morbid changes of a serious nature, by the pressure of the cysts on the surrounding structures, by which these structures are condensed and indurated into tumours of suspicious character, authorising extirpation.

7. *Pancreatic Sarcoma of the Mamma.* Mr. Abernethy states that this tumour bears a strong resemblance to the pancreas; but this, of course, gives us no exact idea of the intimate structure of the morbid growth, since it is very improbable that there should be any thing more than an external resemblance between a natural organ and a diseased product. M. Bayle describes this tumour under the title of "*corps febreux*," and informs us that it presents itself in three different states:—1st, fleshy —2d, fibro-cartilaginous—3tio, osseous. Cases of this kind are related by Mr. Abernethy and Sir Astley Cooper, the latter denominating it the "*simple chronic tumour.*"

8. *Adipose Tumour of the Mamma.* The nature of this species of tumour is easily ascertained by the peculiar lubricity of the swelling and its equable softness.

9. *Fungus Hœmatodes.* Hey and Cooper have proved, beyond a doubt, that this terrible disease does not spare the human mamma; but it is not so certain that the true encephaloid or cerebriform tumour has been found in the organ in question.

10. *Carcinoma Mammœ.* This disease, too familiar to practitioners, and too destructive of female life, is divided by Dr. C. into two species —c. tuberculosum and c. œdematodes. The characters of the first are well known:—Stony hardness, irregularity of surface, insensibility to pressure, and lancinating pains, in the early stages:—When ulceration has taken place, the ulcer has an excavated surface, hard, everted edges, sometimes firm cauliflower excrescences. The second species is more alarming in appearance, and more rapid in its progress. It seems to be irremediable by physic or surgery, unless taken in a very early stage indeed. Justamond, Boyer, and others, have described it. Our author has related two cases in illustration of this terrible malady. We shall present a rapid sketch of one of these.

*Case.* J. Mitchel, aged 31. Her left breast swollen and very hard, but it pitted on long-continued pressure. The left side of the trunk and left upper extremity œdematous—dry cough, dyspnœa, darting pains in the back part of the mamma and under the scapula, impaired appetite, thirst, scanty urine. The swelling of the mamma had come on after exposure to wet and cold, while perspiring, three months previously. Leeches and various remedies were employed; but the disease increased, and she died in twelve days after admission into the hospital. On dissection, the skin was found to be thickened, hard, and filled with a pale fluid, which did not flow out rapidly. The mammary gland was very compact and hard, approaching to that of cartilage. A good deal of serous fluid could be pressed from its substance. The pectoralis major was pale, and of ligamento-fibrous texture. The glands in the axilla were nearly as hard as cartilage. There were 40 ounces of serum in the right side of the chest. The lungs were sound.

A remedy for cancer is yet to be discovered; and even the extirpation, in an early stage, is doubted by some as a certain cure. Baron Boyer says he only saw four or five cases of permanent recovery in one hundred cases or more, where he operated! In all the others the disease re-appeared after a longer or shorter interval, and the patients died. Dr. C. thinks it probable that, in many of these cases, the patients enjoyed an interval of immunity, which proved an adequate reward for the pain and danger of the operation. But even the saving of four or five per cent. of human life, by an early operation, is a justification of the attempt. We hope and believe that the experience of the surgical profession, in this country, would raise the proportion of permanent cures above that which is here stated.

We think the foregoing succinct and luminous classification of mammary diseases will prove useful to the practitioner, and peculiarly so to the surgical teacher.

---

### 16. LARGE DOSES OF ARSENIC IN CHOREA.

The following case, recorded by Dr. James Fountain, of York Town, New York, is very extraordinary, on account of the immense quantity of arsenic exhibited.

The patient was a young lady, of 14 years of age, of very sanguineous temperament, yet of delicate constitution, who had previously enjoyed good health, and had menstruated regularly for some time. The catamenia became suppressed in July, 1826, and Dr. Mead, the family physician, treated the defect by a system of mild tonics, till the 13th September following, when Dr. Fountain was called in. On the 13th, she began to shew symptoms of chorea, and Dr. F. advised an emetic, to be followed by bark and steel, "*ad libitum.*" She was also ordered aloes, myrrh, and assafœtida. *Sept.* 21. She was worse in all respects—pulse 95—tongue clean—strength declining—"spasms frightfully increased"—articulation interrupted—deglutition difficult—mind alienated—violent screaming—hair dishevelled—"in short, exhibiting the most melancholy and heart-rending picture that can be imagined."

Dr. Fountain now determined on a more vigorous course than that hitherto pursued; and accordingly, on the evening of the 21st September, the young lady was ordered to take ten drops of arsenical solution every two hours; and by the 25th she had taken 300 drops? By this time, "the vascular action had become intense," and the pulse was quickened to 120 in the minute. The spasms, however, had considerably abated, and the patient had enjoyed some sleep. Arsenical pills were now directed to be taken, "for a change." On the 26th the spasms ceased entirely. 27th, was comfortable, but gloomy and suspicious. The arsenical pills were decreased in number. The medicine, however, appears to have been continued till the 2nd October, when it was left off, "the nervous irritation having been supplanted by vascular action." Some laxative medicine was exhibited, and by the 10th October, the lady was perfectly well. No swelling of the face was produced, "and neither dropsy, consumption, nor rheumatism have yet appeared."

To practitioners in this, and in most other countries, these doses of arsenic must appear prodigious. The medicine is certainly one of the most powerful tonics in nature, and may be given much more freely than is generally done, without danger. But we should be extremely loth to try such quantities as Dr. Fountain ventured on in the above case. We have found pain in the stomach, puffiness under the eyes, and tensive pulsating pain in the head, produced by much smaller doses than those which were given in the foregoing case.—*N. York Med. and Phys. Journ.*

---

### 17. DESTRUCTION OF ARTICULATING CARTILAGES.

Under the title of "Usure des Cartilages Articulaires," M. Cruveilheir has described a severe complaint of the joints, which is often confounded with gout, rheumatism, white swelling, &c. and treated by means which are useless, if not injurious. This destruction, erosion, or wearing away of the cartilages, is one of the worst effects of inflammation of the synovial membrane of the joints. This effect continues after its cause has ceased to operate; and then the patient experiences a peculiar rigidity of the articulation, with a feeling, and even a hearing of cracking in the joint, on taking exercise, together with pain, of greater or less severity. This crepitus, this rigidity, these pains are particularly felt when the patient first gets up in the morning, and begins to move the joint. Our author has known cases of this kind treated, for a long period, with leeches, blisters, setons, &c. all of which failed—and even amputation had been proposed in some cases.

M. Cruveilheir attended a lady who, after a severe labour, was seized with articular rheumatism, which travelled successively over all the joints. The pains at length diminished, but did not entirely subside. The patient, without experiencing any new attack of rheumatism, perceived her knee-joints to be gradually getting stiff and painful; and she could feel and hear a crepitus whenever she attempted to walk, or even to turn herself in bed. Soon after this, the hip-joints, the shoulders, the elbows, wrists, and even the metacarpal joints were affected in the same way. Various means were used, without effect, and a surgeon had placed extensive moxas on each side of one of her knees, which penetrated to the fibrous tissues, and produced a copious suppuration that continued some considerable time. The patient thought herself relieved, and consented to a similar application to the other knee. But after three months' sufferings in this way, the joints were found by our author as stiff as ever, and also as painful as before. The constitution had now suffered much from the local irritation. Daily motion was now tried; but this augmented the disease in all respects. He found himself on the wrong track, and quickly abandoned this plan. For a long time he hoped that anchylosis would put a period to this lady's sufferings; but in this expectation he was disappointed. One day, on accurate examination, he found that the joints on being moved conveyed to the ear and hand of the surgeon the crepitus above described. M. Cruveilheir then immediately recognized the nature of the malady as obliteration of the cartilages. For this state, there is no known remedy; but as M. C. conceives that it is the result of inflammation of the synovial membrane, the means of arresting the progress of the disease consist, of course, in rest, and in checking the phlogosis of the said membrane. Two very illustrative dissections are given of this obliteration of the cartilages. In one, the disease only affected the knee-joint—in the other, the hip-joint, the elbow, and the articulation of the lower jaw were the seat of the lesion.—*Bibliotheque Med. Janvier*, 1827.

### 18. POISONOUS MATTER IN OFFAL.

[Mr. Brodie.]

In the April Number of the *Med. and Phys. Journ.* Mr. Brodie has stated three cases where a remarkable train of local phenomena followed the handling of offal. We shall give a summary view of these cases.

*Case* 1. A healthy young man cut his fore-finger, while engaged in feeding dogs with sheep's offal. The wound healed in two or three days; but then the end of the finger was found to be inflamed and swollen, as far as the second joint—extending slowly over the first phalanx—up the outside of the hand, as high as the wrist, then downward over the middle finger—again upwards on the palm of the hand to the wrist—and lastly downwards over the whole of the ring-finger, and the first phalanx of the little finger. The inflammation was marked by deep redness of the skin, with slight tumefaction and tenderness. His general health was unaffected. The complaint ran a course of six weeks, and then disappeared without any medical assistance.

*Case* 2. A cook at an hotel, scratched his finger with the extremity of a rib, while eviscerating a hare. In two or three days inflammation occurred, and extended up the finger to the back of the hand, and thence downwards over the adjoining finger, and so on, leaving one part as it invaded another. It was three weeks after the accident when Mr. Brodie saw the patient. The inflamed parts were of a crimson redness, and rather painful and tender on being handled. The health was unaffected. Leeches had been several times applied, but without benefit. Mr. B. prescribed some local applications, which appeared to do little good. The oxymuriate of mercury, in doses of an eighth of a grain, was then given twice a day. In three weeks the redness and swelling had much abated; but there was considerable pain in the hand, extending along the fore-finger. One drachm of the powder of sarsaparilla was ordered thrice a day, in addition to the oxymuriate. In a week of this treatment, the inflammation had nearly disappeared, a numbness and pain remaining, which extended up to the shoulder. This continued for two weeks longer, and then subsided.

*Case* 3. A woman pricked the middle-finger with a splinter of bone, while cleaning the inside of a hare previously eviscerated. On the following day there was slight inflammation, which increased during the succeeding week, attended with pain and sleeplessness. One week after the accident, Mr. Brodie saw the patient, the whole of the middle-finger being then inflamed and swollen, the skin tense and shining. The patient complained of tingling and throbbing pain, which prevented sleep; but there was little or no disturbance of the general system. One-eighth of a grain of oxymuriate of mercury was prescribed, bis die, and the application of a poultice. In eight or ten days the inflammation was checked, and soon afterwards disappeared.

We think that these cases go to shew that there is absorption of a morbid poison or septic principle, analogous to what we see in dissection wounds, though on a minor scale of virulence.

### 19. BROUSSAIS ON ASTHMA.

M. Bonnez, assistant surgeon of the 10th regiment of Chasseurs, in garrison at Libourne, aged 36 years, had the imprudence to bathe in a river, after a hearty dinner, on the 18th of July. In the middle of the night he was seized with general malaise, succeeded next day by fever, head-ache, coryza, and cough. During the night of the 19th he had no rest. On the 20th the phenomena changed into a complete attack of convulsive asthma, (the second paroxysm which he had experienced, the first being three years before) and he then sent for his medical colleague. Ten ounces of blood were taken from the arm, and pediluvia applied to the feet. By these means the symptoms were relieved, and the night of the 20th was spent less miserably. 21st, the paroxysm returned, and continued till near the evening. The anhelation this day was very distressing, and an antispasmodic julep was ordered, which augmented the dyspnœa, and brought on another paroxysm of asthma. The night was spent in a state of agitation. 23rd, Ipecacuan had been taken in small doses, and also castor; but the paroxysm returned this day, with as much violence as before. 24th, The asthmatic paroxysm came on at the usual hour, six in the evening. On the 25th, Dr. Bagard, was called in, and found the patient with the following phenomena:—dejected countenance—eyes sunk—breathing short—pulse small and quick—tongue coated—great tenderness at the epigastrium—oppression under the sternum—abdomen rather tense—urine scanty and high-coloured. Sixteen leeches were applied to the sternum and epigastrium, the bites to be encouraged by cataplasms—very low diet—diluents—lavements. The succeeding paroxysm (26th) was very much milder, being only a simple dyspnœa. 27. When Dr. B. visited his patient, the latter observed that he was quite well, and had a strong desire for food. But it was evident that the patient was not well. At the usual hour, the dyspnœa returned, accompanied by some cough. 28th, Felt very well all day, till six o'clock, when the dyspnœa returned as usual. Dr. B. now being struck with the periodicity of the complaint, and seeing nothing wrong with the digestive organs, prescribed the sulphate of quinine, in doses of three grains every three hours. The next paroxysm was prevented. The remedy was continued for three days, and the patient was free from complaint. On the 6th August, however, when M. Bonnez though thimself in complete security, he was suddenly seized with pain in one side of the chest, with fever, cough, head-ache, &c. Cupping-glasses were applied to the side, and afterwards a large blister. But these means were of no avail. The symptoms became exasperated, and the sputa sanguinolent. When Dr. B. was again called to the patient, he found him with violent head-ache, acute pain in the right side of the thorax, intense fever, full pulse, burning skin, and countenance indicative of despair. It was now evident to Dr. B. that the inflammation had spread from the mucous membrane to the pulmonary parenchyma, and even to the pleura. Dr. B. advised the application of 25 leeches to the chest; but the regimental surgeon protested against any more leeches, and our author took his leave. Two other physicians were called in, and ventured on the abstraction of six ounces of blood from the arm. This made no impression on the complaint, and Dr. Bagard was recalled. He applied 25 leeches to the chest, which, with a blister, completely removed the disease.

*M. Broussais' Remarks.*—Most cases of Asthma depend on some obstacle to the course of the blood; and this obstacle is most commonly a disease of the heart. This, however, is not always the case. A determination (however induced) of blood to the mucous membrane of the lungs, in a sanguineous subject, will often give rise to a paroxysm of asthma, as was the case in the above instance. M. Broussais has known inflammation and irritation in the mucous membrane of the stomach and bowels induce a fit of what is called spasmodic asthma. The Professor ridicules the distinction drawn between dry and humid asthma. Every asthma is dry at the commencement of the paroxysm, and the mucous membrane ultimately throws out a secretion which relieves the vessels of the lungs. In all cases, however, of asthma, M. Broussais avers that there is a congestion of blood in the vessels of the membrane lining the bronchia and air-cells, and that this should be looked upon as the proximate or immediate cause of the phenomena, and treated accordingly.—*Journ. de la Med. Physiol.*

### 20. ABDOMINAL TUMOURS.

At a late sitting of the Royal Academy of Medicine, in Paris, M. Lisfranc related the particulars of a very curious case, which we shall briefly notice in this place. A female had a fluctuating tumour in the abdomen, which caused such an enlargement of that region, that she was thought by her neighbours to be pregnant. She was examined by several surgeons of eminence, who recognized the tumour, but none of them would attempt to draw off the contents. One day, while she was straining to make water, a considerable quantity of a yellowish mucous fluid was discharged, and the size of the abdomen suddenly diminished. She now fell into a state of syncope, and M. Lisfranc was called to her assistance. He saw the cause, and placing her in bed with the hips elevated, the discharge ceased, and the faintness went off. From time to time, the woman was raised to the perpendicular posture, and then the discharge by the urethra returned. In the course of a month the abdomen was reduced to its natural size, and at present, there is only a small, hard, and globular tumour, the size of one's fist, situated in the left iliac region. It has remained in this state for more than two years. There is, however, a slight discharge of whitish fluid from the bladder, which is supposed to come from this cyst. The woman is in good health.

M. Emery related also the case of Madame N. who, five years ago, was delivered of a child. Soon after the pregnancy had commenced, her abdomen became greatly distended, so, that at the end of three months, she was as large as a woman at the full period of utero-gestation. She went on, however, to the full term, and was safely delivered. Three months after her accouchment, M. Emery was obliged to perform paracentesis abdominis, when twenty-four pints of a fluid, resembling molasses, were drawn off. In the course of the three succeeding years, the operation was seven times repeated. The sixth time the paracentesis was performed, the fluid was of a puriform appearance, with albuminous flocculi. The patient was now put on a course of diuretics, and the nitrate of potash was carried to the dose of half an ounce *per diem.* After the seventh operation, the patient passed *per vias urinarias,* a large quantity of pellucid water, amounting to twelve pints in 48 hours. This phenomenon recurred every two months, for more than a year. She is now three months pregnant, during which paracentesis has been once performed, and will soon again be necessary.

M. Lisfranc was of opinion that surgeons were much too timid in opening or injecting encysted tumours of the abdomen. He cited two cases from Le Dran, where the operation was performed with success. M. Margolin was, however, of a different way of thinking. He believed that surgeons could not be too cautious how they meddled with these kinds of tumours. He mentioned the case of a lady whom he and M. Bayle had attended. She had already undergone the operation of paracentesis thrice, and was extremely anxious to be cured by some means or other. He and M. Bayle therefore injected into the cyst, two litres of barley-water with honey. Two hours after the injection a most tremendous peritonitis supervened, which resisted all remedies and quickly destroyed the patient,—*Revue Med. Fevrier.*

We shall probably be able to state, in some part of this number, a case of a very extraordinary kind, which lately occurred in the person of a lady residing in Essex, and whom we saw several times during the last 18 months. The case bears intimately on the present subject.

---

### 21. POISONING BY ARSENIC AND LAUDANUM.

Mr. Scott, of Newington Causeway, has stated a case in the last Number of the Medical and Physical Journal, the object of which will be apparent in the sequel.

A young woman, aged 16 years, swallowed, by her own account, "fifteen penny-worth of laudanum and half a tea-cupful of arsenic," of which she died in six hours. Mr. Scott did not arrive till near the fatal period, and found her writhing under great torture. She had vomited severely for two hours; and dark offensive motions had been frequently discharged from the bowels. Mr. S. proceeded to work the stomach pump, and two quarts of warm water were twice thrown in and withdrawn by means of that instrument. Very little indeed of the arsenic was dislodged, and as the patient was now in articulo mortis, the process was discontinued. In ten minutes more she expired. The stomach, on being opened, was found to contain about 20 ounces of fluid, which had been injected by the pump. On removing this fluid, the surface of the organ presented a universal vermilion blush, with brownish red patches scattered here and there, chiefly in the pyloric portion. These patches were somewhat pulpy—loose in texture—gellatinous in appearance—and more glossy than the surrounding parts—in fine, "they were portions of the mucous membrane in a state of disorganization," and might be easily detached by pinching with the finger and thumb, leaving the muscular coat denuded. "*Near the extremity of the stomach, lay two masses of powdered arsenic, enveloped in a sort of reddish jelly*, which doubtless consisted of the mucous membrane disorganized by the contact of the poison." These were scraped off with a spoon—the quantity might be about half an ounce.

Here Mr. S. properly invites the attention of the profession to the fact that, vomiting, assisted by copious dilution, *during two hours*, had not detached the arsenical powder from the surface of the stomach; and so entangled was the poison with the softened mucous membrane, that he believes no action of the organ itself could have separated it. The injection of a strong current, by means of the syringe, had even failed to detach the mineral. From this Mr. S. concludes, and we think with justice, that the efficacy of emetics, in dislodging arsenic from the stomach, is confined to a limited time after the poison has been swallowed, though that limit is not yet ascertained, being probably influenced by the quantity swallowed, by the quality and volume of ingesta, and other accidental circumstances. In the absence of all positive information, on this point, Mr. Scott queries, would it not be the safest practice to resort, at once, to the stomach-pump in these cases? Mr. S. here alludes to the superiority of the stomach-pump over any apparatus acting on the principle of the syphon, in consequence of the great propelling power of the former, by which arsenic might be dislodged from localities in the stomach. He might have added, that the activity of the suction power (speaking vulgarly) also gives the pump an advantage over the syphon.

---

### 22. INTERMITTENT RHEUMATIC FEVER.

The patient was Dr. Costa, Physician to the Great Lazaret of the Eastern Pyrennees. In the afternoon of February 1st, 1826, Dr. C. was seized with a violent cold chill along the spine, pains in the limbs and loins, cramps, &c. In a couple of hours, the cold chills ceased, but the pain in the back increased, and extended to the shoulders and neck, so that he could scarcely use the least flexion or rotation of the head. In the evening the pain became excessive in the back part of the head, and the night was spent in great agitation. At six in the morning, of Feb. 2d, the pain diminished, and some sleep was obtained, followed by perspiration, and a complete solution of the paroxysm. The Doctor now considered himself as cured by the critical sweat, and he resumed his usual avocations. At three o'clock, however, the enemy returned, in the form of a cold chill, then heat, fever, and all the usual symptoms of re-action, accompanied by intense pain from the loins to the occipito-frontalis muscle. At each pulsation of the heart, the pain was so excessive in the integuments of the head, as almost to drive the patient mad; yet there was neither redness, or tumefaction of the parts, there being only an increase of heat. Strong pressure diminished the pain. His eyes were red and prominent—his countenance flushed—his whole body burning. His stomach was undisturbed—there was no tenderness on strong pressure at the epigastrium—no thirst—tongue pale and moist, but slightly furred—in short, there was no function disturbed but that of sensation—his whole sufferings were in the integuments of the head. The paroxysm continued till three o'clock next morning, when he fell into a sleep, exhausted by the pain. He awoke free from all complaint except weariness, and stiffness of the muscles. Instead of profiting by the intermission which now took place, the Doctor did nothing, except taking some lemonade and a glyster. At 4 o'clock on the 3d of February, the paroxysm returned, with more violence, if possible, than ever, and ran the same course as before. He was now grown wiser by experience, and as soon as the intermission took place, he had recourse to the sulphate of quinine, of which he took twelve grains. The next paroxysm was four hours later than usual, and the symptoms were infinitely mitigated. Another medication of the quinine put an end to the complaint, with a very trifling memento of the phenomena, for one or two days more.

We question whether the adjunct "*rheumatic*" is applicable to the above case. It was, in fact, one of the various forms which ague assumes, in different localities, and in different constitutions. It is evident that depletion would have been ineffectual, if not prejudicial in

Dr. Costa's case, and that the quinine was the best remedy. Yet we daily see medical men have recourse to profuse bleedings, local or general, during the paroxysms of these periodical diseases, without making the least impression on the complaint.—*Bibliotheque Medicale.*

---

### 23. NERVOUS ANASTOMOSES OF THE GREAT SYMPATHETIC WITH THE FIFTH PAIR OF NERVES.*

The difficulty of accounting for tic douloureux and other painful affections of the face, as arising from irritation in the stomach and bowels, for instance, has often been acknowledged; and the vidian nerve has been considered as the most demonstrable medium of communication between the great sympathetic and the fifth pair. Hence was inferred the general inutility of dividing the branches of the fifth pair in tic douloureux, as the connexion by the vidian nerve was still left open. But supposing that the vidian nerve was cut, (which would be a pretty difficult operation,) the connexion between the nerves of the stomach and those of the face would not be the less free. We consider the *brain* as the medium of all nervous sympathy—and unless the surgeon can cut off *that* channel of communication, he will fail, we apprehend, in all his neurotomical operations for the cure of painful affections of the face, or of any other part of the body, dependent on visceral irritation.

Professor Jacobson, of Copenhagen, has, however, published an account of an anastomosis between the sympathetic and trifacial nerves, to which he appears to attach great importance. As M. Breschet, in his notes on this discovery, has not only thrown doubts on Jacobson's descriptions, but otherwise lessened their importance, supposing them to be correct, we shall give only a very brief account of this Jacobsonian anastomosis.

The glosso-pharyngeal nerve issues from the cranium by a particular canal, formed principally by the petrous portion of the temporal bone, and separated from the foramen lacerum poster. by a fibro-cartilaginous production. In examining this canal, a considerable fossa may be observed, close to which, a small canal branches off and leads to the cavity of the tympanum. In the above fossa the nerve forms a swelling or ganglion (un renflement ou ganglion) described by Andersch, and it is from this ganglion that the anastomosing branch, the subject of the present memoir, proceeds. Arrived at the cavity of the tympanum, it divides into three filaments—a superior, anterior, and inferior. It is principally by means of the first two that the anastomosis with the fifth pair is formed—the third filament unites with the sympathetic nerve, and distributes some minute threads to the membrane surrounding the opening of the eustachian tube. The *superior* filament is the most considerable, and traversing the promontory, directs its course towards the internal angle of the foramen ovale, where it enters a canal—passes under the internal muscle of the malleus—and makes its exit by an opening in the sulcus for the cranial branch of the pterygoid nerve, with

---

* Repertoire, No. 4, p. 107—204. By Professor Jacobson, of Copenhagen.

which it ultimately unites,* and both proceed to join the supra-maxil lary nerve, or the spheno-palatine ganglion of Meckel.

The *anterior* filament having gone round the cavity of the tympanum, enters a canal formed in the parietes of the foramen caroticum, or where this parietal canal is wanting, the filament glides behind the eustachian tube, and enters the pterygoid canal, and passes on to the spheno-palatine ganglion of Meckel. The *third* filament, leaving the others at the end of the common canal, glides along the inferior part of the tympanum, following the posterior parietes of the tube to its opening, and there terminating in a leash of filaments to the back part of the palate. This *third* filament gives off a branch that communicates with the sympathetic through the foramen caroticum.

This anastomosis has been observed by our author in a great number of the mammalia, and he ventures to affirm that it exists in nearly all of them.

We shall dwell no longer on this anastomosis, because we do not think that it explains any sympathetic connexion between distant parts, either physiologically or pathologically.

Viewing the brain and spinal marrow as the two extremities of one substance—one organ, it is quite preposterous to hunt after minute filamentary communications between particular nerves, when their origin or sensorium commune is the same. If the highest function of the brain itself, the exercise of the intellect, sympathises rapidly and powerfully with a filament of nerve, which may be irritated in the stomach or other chylopoietic viscera, where is the wonder that subordinate functions of the brain, or of any of the nerves issuing from the brain, should also be sympathetically affected?

At all events, the connexion of the grand sympathetic with the greater number of the cerebral nerves has been traced by many anatomists, and especially by C. A. Bock. For example, he has proved the connexion of the *trisplanchnic* nerve with the *fifth pair*. 1mo, by the ganglion of Gasser, which is united to the sympathetic by very delicate filaments—2do, by the carotid ganglion, which communicates with the first branch of the fifth pair—3tio, by the same ganglion, which sends two filaments to the ciliary ganglion—4to, by the vidian nerve—5to, a nervous branch of the soft plexus of the great sympathetic unites with the maxillary ganglion of the lingual branch of the fifth pair. We need hardly say that when the connexion between the sympathetic and the fifth pair is fairly established, we can have no doubt that all the nerves of the body are in communication with each other.

---

### 23. CLIMATE OF MADEIRA IN CASES OF PHTHISIS.**

Medical men are often led to sanction the emigration of a consumptive patient to a warmer climate than that of England, when their

* "Ce n'est qu'apres un trajet plus ou moins long, qu'il est entierement uni," &c. By omitting the word "till," the translator of this paper in the Lancet has rendered the passage complete nonsense. "It is not after a certain course that it is entirely united," &c.—*Lancet*, April 21st, 1827.

** Dr. Renton. Ed. Journal, April, 1827.

judgment tells them that no good is to be expected from that measure. The eagerness of the patient and friends to grasp at a possible chance of saving or prolonging life—the fear of being suspected of mercenary motives in recommending the patient to remain at home—and the uncertainty as to the actual amount of organic disease in the lungs—these are circumstances that induce the medical adviser not to throw the weight of his opinion against the projected emigration. There are many medical men too, who have no personal experience of the effects of a warm climate on pulmonary consumption ; and who have a vague idea that, as the cold, variable, damp, and gloomy atmosphere of England tends to the production of the disease, so the bright skies, and the higher and steadier range of temperature in Italy, Portugal, or Madeira, should have a contrary tendency. The whole question, however, hinges on the nature and the amount of pulmonary disease ; and if there was no other good to be derived from the late attempts to improve the diagnosis of thoracic affections, than what would accrue to patient and practitioner, when the question of emigration is raised, the study of auscultation might still deserve cultivation.

Those who reside in Madeira, Lisbon, Nice, and other spots supposed to be favourable to consumptive patients, have daily evidence, both from the oral statements of the victims themselves, and the written documents which accompany them, that unfounded and flattering hopes have been held out to breathless and almost lungless wretches, who are exiled from friends and sacred home, first to undergo the misery of a sea-voyage or long journey, and then to end their days among strangers, far from the consolation and attention of parental or fraternal affection.

Dr. Renton has drawn a mournful but correct picture of the effects of this rash and unpardonable medical recommendation which induces or sanctions the removal to Madeira, under circumstances where no rational hope of recovery can or should be entertained. Thus, of 47 cases of phthisis sent to Madeira, 32 died within six months after their arrival—six died on a second winter's trial of the climate—six died after returning to England—while the fate of three remained unascertained, though the probability was, that they too died, after undergoing all the trouble, expense, and fatigue of the voyage out and home!

Under what circumstances then is the medical adviser justified in recommending Madeira or other warm climate? Only, we conceive, where there is a tendency to pulmonary hæmorrhage from delicacy of pulmonary structure, incipient tubercles, or condensations of lung to no great extent. Where tubercles have arrived at a large size, and especially when they have softened down, and their contents are making their way through the bronchia, in the form of purulent expectoration, with all its usual attendants, emaciation, cough, colliquative diarrhœa, hectic fever, morning perspirations, &c. it is cruel, unjust, and unpardonable to sanction, much more to recommend a voyage to Madeira, or a journey to France or Italy. But it is not to be denied, that there are cases where the nature, or rather the source of the purulent expectoration is doubtful—and where a presumption that it comes from the mucous membrane, and not from tubercular excavations, may render the recommendation of the medical adviser, as to emigration, justifiable, and indeed humane. It is in such cases, that the utmost pains should be taken to ascertain the precise condition of the thoracic organs; and we fearlessly maintain that this cannot *always* be done, without the aid of auscultation and percussion. We know, indeed, that some medical men, who have unlimited confidence in their own powers of discrimination, will decide upon such cases, after half a dozen of common-place questions, without a single examination of the naked thorax. But whether they are invariably correct in their diagnoses, we will not venture to say. We suspect that Dr. Renton could solve this query; for he informs us that he examined the bodies of 15 patients who had been sent out to Madeira, and that—"in some of them the pulmonary symptoms were stated to be *merely secondary*, and the *liver* denounced as the offender in chief;" yet in only *one* instance was this organ found affected in structure, and it was then tangibly and unequivocally enlarged. It is almost incredible the mischief that is done in this country, by mistaking real pulmonary *disease* for symptomatic pulmonary *disorder*, occasioned by hepatic or gastric affection! Many of the most melancholy cases that come under our observation, are those, where the thoracic symptoms have been despised, and where the hepatic derangement has absorbed the whole attention of the medical practitioner, till the structure of the lungs has been changed by inflammation into irremediable formations. We have reason to believe that we have awakened the attention of the profession to this delusion, and that some lives will be saved in consequence.

In conclusion, we agree with Dr. Renton, that "great and lasting benefit is to be derived from even a temporary residence in the climate of Madeira, in cases where pulmonary disease is *merely threatened*, or where strong family predisposition exists." But, even in such cases, the greatest attention is necessary to avert from the delicate organ every accession of inflammatory excitement, by diet, medicine, and cloathing.

P. S. We have had intelligence from several medical invalids, who have been recently trying various parts of the Continent, that the situation of Hieres, in the South of France, is, upon the whole, the best adapted for pulmonary affections.

---

### 25. M. BROUSSAIS ON TETANUS.

The Professor of the Val de Grace has recently published a memoir on tetanus, by M. Lassere, a physician of Dordogne, in which the author of the new physiological doctrine strongly advocates the practice of local depletion in tetanus, as the only measure that promises any thing like general success in this formidable malady.

Dr. Lassere has met with five cases of tetanus within these few years —the first four of which were saved by general, and more especially local depletion along the spine, the epigastrium, and the muscles which were the seat of spasm. One of these cases was traumatic tetanus, too, which is still more dangerous than the idiopathic. Of this case we shall take a short notice.

Margaret Fouillard, had her heel wounded by a blunt piece of iron, the injury, however, being so trifling as not to prevent her from attending to her domestic concerns. Two days afterwards, she felt pains shooting up the thigh of that side,—and, ultimately the back, which became stiff. On the tenth day after the accident our author was called to the patient, the whole trunk of whose body, and also the lower extremities, were rigid as a board. Trismus was slight; but the spinal column was the seat of excruciating pain. Her bowels had not acted for some days—pulse small and tight—sense of suffocation. On examining the seat of the original injury, Dr. L. found the parts tender on pressure, though without any swelling. He made a deep crucial incision, which occasioned a profuse hæmorrhage. Thirty leeches were applied to the lumbar region, and then a large cataplasm. Opium was also given every two or three hours through the night. The next morning the tetanic symptoms were greatly relieved, and the trismus had disappeared. Warm baths, purgatives, and fomentations to the injured heel completed the cure.

The above was the slightest of all the five cases—the others requiring great and repeated local depletions by leeches from the spine and epigastrium. In some of these cases, it was observed that opium not only disturbed the head, but seemed to induce or accelerate inflammation of the entero-gastric mucous membrane.

M. Broussais, in his comments on these cases, ridicules the idea of treating tetanus, as a nervous or spasmodic affection, by opium, antispasmodics, mercury, cold and warm baths, &c. It must be treated as we would treat arachnitis or spinitis—"that is, by applying leeches along the vertebral column, and along those muscles to which an excess of nervous influence is directed." He strongly censures the administration of opium in this disease. He avers that this medicine excites disorder in the stomach and head, which disorder re-acts on the spinal irritation or inflammation, and, consequently, increases the disease. On the same account, he abstains from all violent purgatives, considering the obstinate constipation as a consequence of the disease, and to be remedied by the removal of its cause. M. Broussais asserts that, treated on these principles, the disease has ceased to be half so formidable as it formerly was in the Val de Grace, and in other places where the physiological doctrine is taught and pursued.—*Journ. de Med. Phys. Fev.*

We believe that the local depletion of Broussais has never been carried to the full extent in this country; and when we reflect that the brain and spinal marrow must be the *immediate* seat of the irritation or inflammation which gives origin to the phenomena of tetanus, we can hardly look with confidence to any remedy which has not a strong tendency to remove this irritation or inflammation. What remedy is more likely to effect this indication than powerful and repeated local depletion from the head and spine, but especially from the latter?

